# Expression and Immunostaining Analyses Suggest that *Pneumocystis* Primary Homothallism Involves Trophic Cells Displaying Both Plus and Minus Pheromone Receptors

**DOI:** 10.1128/mBio.01145-19

**Published:** 2019-07-09

**Authors:** A. Luraschi, S. Richard, J. M. G. C. F. Almeida, M. Pagni, M. T. Cushion, P. M. Hauser

**Affiliations:** a Institute of Microbiology, University of Lausanne, Lausanne, Switzerland; b Lausanne University Hospital, University of Lausanne, Lausanne, Switzerland; c UCIBIO-REQUIMTE, Faculdade de Ciências e Tecnologia, Universidade Nova de Lisboa, Caparica, Portugal; d Vital-IT Group, SIB Swiss Institute of Bioinformatics, Lausanne, Switzerland; e Department of Internal Medicine, University of Cincinnati College of Medicine, Cincinnati, Ohio, USA; f Veterans Administration Medical Center, University of Cincinnati College of Medicine, Cincinnati, Ohio, USA; Albert Einstein College of Medicine

**Keywords:** homothallism, opportunistic fungi, sexuality

## Abstract

The genus *Pneumocystis* encompasses fungal species that colonize mammals’ lungs with host specificity. Should the host immune system weaken, the fungal species can cause severe pneumonia. The life cycle of these pathogens is poorly known, mainly because an *in vitro* culture method has not been established. Both asexual and sexual cycles would occur. Trophic cells, the predominant forms during infection, could multiply asexually but also enter into a sexual cycle. Comparative genomics revealed a single mating type locus, including plus and minus genes, suggesting that primary homothallism involving self-fertility of each strain is the mode of reproduction of *Pneumocystis* species. We identified and analyzed the expression of the *mam2* and *map3* genes encoding the receptors for plus and minus pheromones using reverse transcriptase PCR, in both infected mice and bronchoalveolar lavage fluid samples from patients with *Pneumocystis* pneumonia. Both receptors were most often concomitantly expressed during infection, revealing that both pheromone-receptor systems are involved in the sexual cycle. The *map3* transcripts were subject to alternative splicing. Using immunostaining, we investigated the presence of the pheromone receptors at the surfaces of *Pneumocystis* cells from a patient. The staining tools were first assessed in Saccharomyces cerevisiae displaying the *Pneumocystis* receptors at their cellular surface. Both receptors were present at the surfaces of the vast majority of the cells that were likely trophic forms. The receptors might have a role in mate recognition and/or postfertilization events. Their presence at the cell surface might facilitate outbreeding versus inbreeding of self-fertile strains.

## Abstract (precis)

The fungi belonging to the genus *Pneumocystis* may cause severe pneumonia in immunocompromised humans, a disease that can be fatal if not treated. This disease is nowadays one of the most frequent invasive fungal infections worldwide. Whole-genome sequencing revealed that the sexuality of these fungi involves a single partner that can self-fertilize. Here, we report that two receptors recognizing specifically excreted pheromones are involved in this self-fertility within infected human lungs. Using fluorescent antibodies binding specifically to these receptors, we observed that most often, the fungal cells display both receptors at their surface. These pheromone-receptor systems might play a role in mate recognition and/or postfertilization events. They constitute an integral part of the *Pneumocystis* obligate sexuality within human lungs, a cycle that is necessary for the dissemination of the fungus to new individuals.

## INTRODUCTION

The genus *Pneumocystis* encompasses fungal species that are extracellular parasites colonizing the lungs of mammals (1, 2). Each species is specific for a single mammalian species, although exceptions exist in rodents (3). The species infecting humans is Pneumocystis jirovecii, while Pneumocystis murina and Pneumocystis carinii infect mice and rats, respectively. Should the human immune system weaken, P. jirovecii can turn into an opportunistic pathogen that causes severe pneumonia, which can be fatal if not treated (*Pneumocystis* pneumonia [PCP]). This disease is nowadays the second most frequent life-threatening invasive fungal infection worldwide, with more than 400,000 annual cases (4).

Despite the importance of PCP, an *in vitro* long-term culture method for *Pneumocystis* organisms has still not been established. Schildgen et al. (5) described a system of coculture with human airway epithelial cells, but to our knowledge, no one has been able to reproduce it so far (6). Consequently, the life cycles of these pathogens have mostly been deduced from microscopic and molecular studies on the model P. carinii, and thus remain hypothetical (7). The genes ensuring several synthesis and assimilation pathways are missing from the *Pneumocystis* genomes, showing that these pathogens are obligate parasites without free-living forms (8–12). Accordingly, their life cycle occurs entirely inside the host’s lungs. It would include both asexual and sexual cycles. The asexual cycle would involve trophic cells that are mostly haploid and predominant during the infection (up to 98% of the cell population; formerly called trophozoites or trophs). These cells are apparently devoid of a cell wall, and might be able to divide by binary fission or endogeny (7). The sexual cycle is thought to involve mating of two trophic cells, generating a zygote that then undergoes meiosis. This process would end with the production of a new cell, the ascus (formerly called cyst), which is surrounded by a thick wall and contains eight daughter cells, the ascospores. Asci are found in the vast majority of human infections, and staining of their wall is used as a diagnostic tool. Asci and/or ascospores are necessary for the transmission of the pathogen to new hosts as aerially transported particles (13, 14).

There are two modes of sexual reproduction among fungi, heterothallism and homothallism (15). Heterothallic fungi need two compatible cells for mating, each expressing transcription factors responsible for the differentiation into a single mating type. The genes encoding these transcription factors are localized in the mating type (*MAT*) locus. Homothallic reproduction includes two modalities. Primary homothallism involves self-fertile strains that harbor the genes for the differentiation into both mating types, in two separated or fused *MAT* loci. Secondary homothallic fungi harbor three *MAT* loci, of which only one is expressed while the other two are silenced. The expressed locus can be exchanged with a silenced one by a mechanism of switching, so that a cell can switch from one mating type to the other mating type. A single *MAT* locus corresponding to a fusion of the minus (M) and plus (P) *MAT* loci was found to be present within each *Pneumocystis* genome (16). This suggested that these species are primary homothallic fungi. This hypothesis was further supported by ascertaining the function of one *Pneumocystis MAT* gene through restoration of sporulation in Schizosaccharomyces pombe, and the concomitant expression of the *MAT* genes during infection (17). The latter observation suggested that *Pneumocystis* sexuality is obligate within the host’s lungs in order to complete the cell cycle and produce asci that are necessary for the dissemination of the fungus.

The sexual reproduction of fungi most often involves signaling through excretion of mating pheromones that are recognized by receptors anchored in the cell membrane. During heterothallic reproduction, a cell of P mating type releases P pheromones that are detected by a cell of M mating type thanks to a receptor specific for the P pheromone (hereafter called P receptor), and vice versa. Genes encoding two pheromone receptors were identified in *Pneumocystis* genomes (16, 18, 19). As far as P. carinii is concerned, the M receptor has been characterized and found to be expressed only in trophic cells (Map3, formerly called Ste3 [18]). On the other hand, the P receptor has not been characterized, but it is annotated in the databases (Mam2, formerly called Ste2 [19]). As far as *P. jirovecii* and P. murina are concerned, candidate genes encoding these receptors have been reported (16, 19), but they were not annotated. Thus, the primary homothallism of *Pneumocystis* species could involve two pheromone-receptor systems. However, both receptors could be expressed in each cell at the same time, or only one of them, thanks to some mechanism. It was also possible that only one of the two receptors was functional, the other one being a relic of an ancestor. The aims of the present study were (i) to identify the *P. jirovecii* and P. murina genes encoding the receptors and characterize the latter, (ii) to investigate the expression of these genes during infection in humans and mice, and (iii) to determine whether only one or both receptors are present on the surfaces of single cells.

(The present work was submitted by A. Luraschi as partial fulfillment of a Ph.D. degree at the Faculty of Biology and Medicine of the University of Lausanne.)

## RESULTS

### Identification of the *P. jirovecii* and P. murina *mam2* and *map3* genes.

Two groups have released the *P. jirovecii* genome sequence (Cissé et al. [9] and Ma et al. [19] [hereafter we call their genome sequences the Cissé assembly and Ma assembly, respectively]). We identified unique Mam2 and Map3 proteins within each of the two *P. jirovecii* proteomes using the sequences of those proteins of P. carinii as homology queries (these proteins were formerly called Ste2 and Ste3). The nucleotide sequences of the two *mam2* genes were identical, whereas those of the two *map3* genes presented several differences (see Fig. S1 in the supplemental material). First, there were three single nucleotide polymorphisms between the two assemblies, one being nonsynonymous (at position 391 in the Cissé assembly [Fig. S1B]). Second, the open reading frame (ORF) predicted in the Ma assembly was 153 bp longer than that in the Cissé assembly, 69 bp upstream and 84 bp downstream. The latter two regions are identical in both assemblies. Thus, there is no stop codon that would be consistent with the shorter protein described in the Cissé assembly, revealing that the latter misses 27 amino acids at the C terminus. Interestingly, for both *mam2* and *map3* genes, the first of the two introns conserved the frame of the ORF without a stop codon, presented the canonical splicing motifs, but was predicted only in the Cissé assembly. Similarly, unique Mam2 and Map3 proteins were identified within the proteome of P. murina, the *mam2* and *map3* genes presenting, respectively, one and two introns each containing a stop codon in the frame of the ORF (Fig. S2B). Consistent with their potential expression, one potential TATA box and one potential cap-signal were identified upstream of the start codon of all the ORFs that we identified (Fig. S3).

10.1128/mBio.01145-19.1 FIG S1 Multiple sequence alignment of *P. jirovecii mam2* (A) and *map3* (B) ORF and genomic gene sequences. Dashes and asterisks indicate gaps and identity, respectively. Introns are highlighted in gray with canonical donor, potential branch site including the branch point A, and acceptor sequences in bold type. The sequences of the two *P. jirovecii* genome assemblies are indicated as Cissé (9) and Ma (19). Single nucleotide polymorphisms between the two assemblies are shown in bold type. The *map3* ORF of the Cissé assembly is shorter than that of the Ma assembly at its two extremities (see text). Pj, *P. jirovecii*; gen, genomic. Download FIG S1, DOCX file, 0.02 MB. Copyright © 2019 Luraschi et al. 2019 Luraschi et al. This is an open-access article distributed under the terms of the Creative Commons Attribution 4.0 International license.

10.1128/mBio.01145-19.2 FIG S2 Sequence alignment of P. murina *mam2* (A) and *map3* (B) ORF and genomic gene sequences. Stop codons within introns are in red. See the legend to Fig. S1 for other details. Pm, P. murina. Download FIG S2, DOCX file, 0.02 MB. Copyright © 2019 Luraschi et al. 2019 Luraschi et al. This is an open-access article distributed under the terms of the Creative Commons Attribution 4.0 International license.

10.1128/mBio.01145-19.3 FIG S3 Potential TATA box and cap signal upstream of *P. jirovecii* and P. murina *mam2* and *map3* genes. Potential TATA boxes are shown with an arrow oriented toward the ORF, and their distance to the start codon of the ORF is given in bps. Potential cap signals are shown in red. The *P. jirovecii map3* ORF of the Ma assembly and its potential promoter elements are shown in blue. Download FIG S3, TIF file, 2.2 MB. Copyright © 2019 Luraschi et al. 2019 Luraschi et al. This is an open-access article distributed under the terms of the Creative Commons Attribution 4.0 International license.

### Characterization of the *P. jirovecii* and P. murina Mam2 and Map3 receptors.

**FIG 1:**
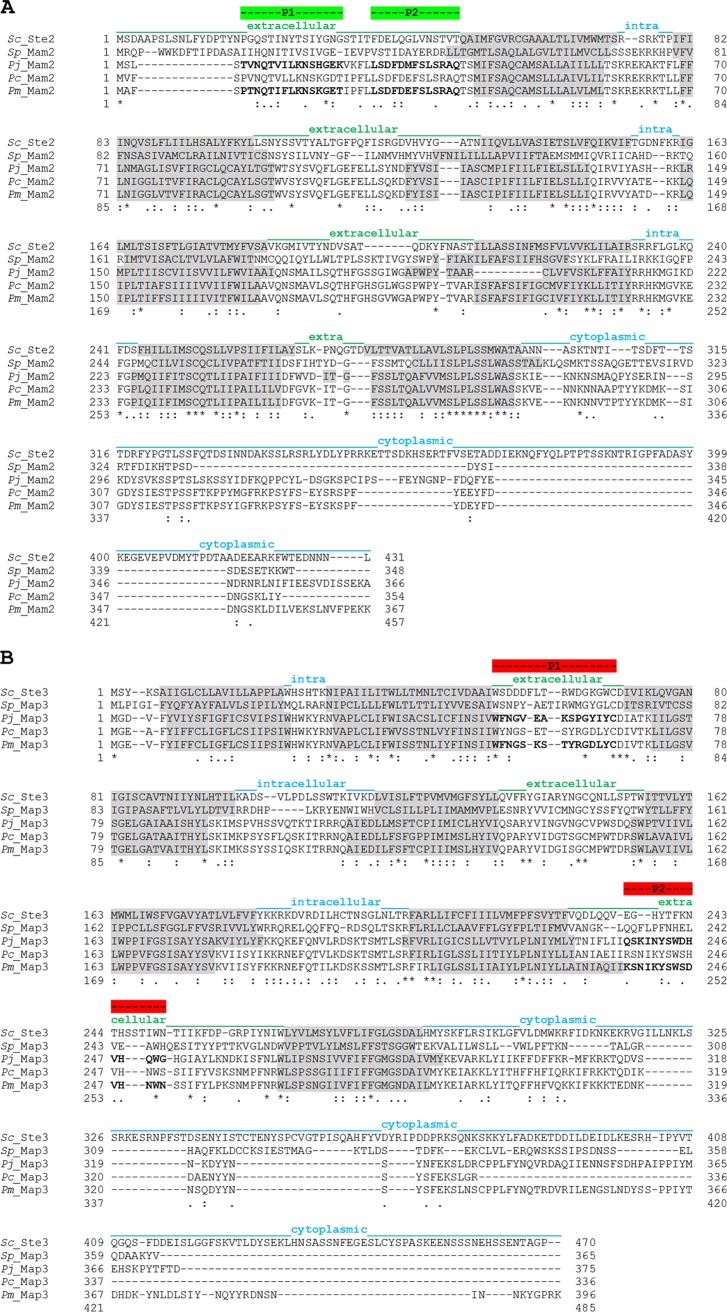
Multiple sequence alignment of Mam2 (A) and Map3 (B) pheromone receptors. The identical, strongly, and weakly conserved residues are indicated by asterisks, colons, and periods, respectively, below the sequence alignment. Dashes indicate gaps to maximize alignment. The transmembrane domains are shown on a gray background. The extracellular, intracellular, and cytoplasmic C-terminal domains are indicated above the sequence alignment. Peptides 1 and 2 used to generate antibodies are shown in green for Mam2 and red for Map3, and the corresponding residues are shown in bold type in *P. jirovecii* and P. murina sequences. (A) Alignment of Mam2 proteins of S. cerevisiae (UniProt ID D6VTK4), S. pombe (Q00619), *P. jirovecii* (L0PDU6, Cissé assembly), P. carinii (A2TJ26), and P. murina (M7P3B3). (B) Alignment of Map3 proteins of S. cerevisiae (P06783), S. pombe (P31397), *P. jirovecii* (L0PBZ8, Cissé assembly), P. carinii (Q9HDG3), and P. murina (M7NMS4). *Sc*, S. cerevisiae; *Sp*, S. pombe; *Pj*, *P. jirovecii*; *Pc*, P. carinii; *Pm*, P. murina.

Multiple sequence alignments of the *Pneumocystis* pheromone receptors we identified with those from relevant fungi are shown in Fig. 1 (the *P. jirovecii* proteins of the Cissé assembly were used because these were studied in the present work). The alignment of the Mam2 receptors revealed that intron 1 in the *P. jirovecii* protein might be an inaccurate prediction because its translation product is present in all other receptors, being identical in the P. carinii and P. murina proteins (positions 199 to 209 of the latter [Fig. 1A]). The alignment of the Map3 receptors revealed that the upstream start of the protein in the Ma assembly might be not used because the protein start of the Cissé assembly aligns consistently with the N termini of the other receptors (Fig. 1B). This alignment also suggested that intron 1 was a correct prediction because its translation product was also absent in the other receptors (between positions 176 and 177 of *P. jirovecii* Map3). The sequence identities among these receptors are given in Table 1. They seem to reflect the phylogenetic distances between the organisms, S. pombe being a member of the Taphrinomycotina subphylum like *Pneumocystis* species, whereas Saccharomyces cerevisiae belongs to the Saccharomycotina subphylum. The orthology of these proteins was further supported by their attribution to the fungal pheromone receptor superfamily Ste2 (pfam02116) or Ste3 (pfam02076) through analysis of the conserved domains. Each of these receptors harbors seven transmembrane domains, strongly suggesting that they are localized within the cell membrane. Consistently, we did not detect any glycosylphosphatidylinositol anchor signals at the ends of these proteins that would be compatible with localization in the wall.

**TABLE 1:** Sequence identity among Mam2 and Map3 receptors of relevant fungi

Receptor	Fungal species	Sequence identity (%) between receptors of fungi			
		P. murina	P. carinii	S. cerevisiae	S. pombe
Mam2	*P. jirovecii*^*a*^	64	64	21	28
	P. murina		89	22	30
	P. carinii			21	30
	S. cerevisiae^*b*^				22

Map3	*P. jirovecii*^*a*^	61	57	21	23
	P. murina		73	21	21
	P. carinii			19	20
	S. cerevisiae^*c*^				20

a The *P. jirovecii* receptors of the Cissé assembly were analyzed. The receptors of the Ma assembly had identities 1 or 2% lower than those of the Cissé assembly, except for Map3, which was 6% lower compared to P. carinii because of 27 additional residues at the C terminus.

b S. cerevisiae Ste2 corresponding to Mam2.

c S. cerevisiae Ste3 corresponding to Map3.

### The *Pneumocystis mam2* and *map3* genes are often expressed concomitantly during infection.

**FIG 2:**
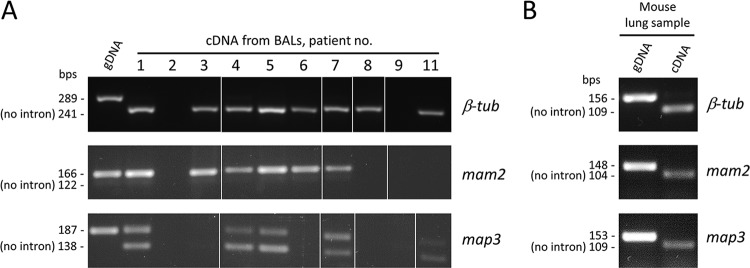
Amplification of the *Pneumocystis mam2*, *map3*, and β-*tub* transcripts by reverse transcriptase PCR. Analysis of cDNAs obtained from 10 BAL fluid samples from 10 patients with PCP (A) and from a sample from infected mouse lungs (B). Genomic DNA from patient 3 or from infected mouse lungs was used as a positive control. The PCR products were of the expected sizes shown next to the bands. The PCR products were obtained from different experiments, as shown by the white strips between pictures. bps, bases pairs; gDNA, genomic DNA.

The expression of the 2 pheromone receptors could be necessary for the occurrence of the sexual cycle that is obligate during *Pneumocystis* pneumonia. However, the possibility of expression of only one of the receptors at the population level by some mechanism was not excluded. To investigate this issue in humans, we used reverse transcriptase PCR analysis of total RNAs extracted from 10 bronchoalveolar lavage (BAL) fluid samples from 10 patients with PCP. We previously analyzed these samples for the expression of the *MAT* genes and ensured that the RNAs did not contain genomic DNA by (i) the lack of amplification in the absence of reverse transcription and (ii) the lack of intron in the PCR product from the unrelated gene encoding β-tubulin (β*-tub*) (17). For a control in the experiments of the present study, we repeated the latter amplification and consistently obtained the same results (Fig. 2A). Of the 10 patients, 4 were positive for the expression of both *P. jirovecii mam2* and *map3* genes, 3 for only one receptor gene, and 3 were negative for both receptor genes (Fig. 2A) (the presence of 2 PCR products for *map3* in each patient is analyzed here below). The latter three patients were also negative for some or all *MAT* and β*-tub* transcripts (Table 2), suggesting that RNA degradation may have occurred, possibly during the uncontrolled period between collection of the samples from the patients and their arrival in our laboratory. The three patients positive for only one receptor might reflect low expression resulting from collection of the BAL fluid sample not at the peak of expression, possibly at an early or late stage of infection. This low expression might also result from the treatment against *P. jirovecii* received by the patient or from a low proportion of trophic cells that presumably express the receptors.

**TABLE 2:** Reverse transcriptase PCR amplification of *Pneumocystis* transcripts from BAL fluid samples from 10 patients with PCP and from infected mouse lungs

Source of *Pneumocystis* transcript	PCR amplification result^*a*^					
	β*-tub*^*b*^	*MAT* transcription factors			Pheromone receptors	
		*matMc*	*matMi*	*matPi*	*mam2*	*map3*
*P. jirovecii* cDNA from patient no.:						
1	+	**+**	**+**	**+**	**+**	**+**
2	−	−	−	−	−	−
3	**+**	**−**	**+**	**−**	**+**	**−**
4	**+**	**+**	**+**	**+**	**+**	**+**
5	**+**	**+**	**+**	**+**	**+**	**+**
6	**+**	**−**	**−**	**+**	**+**	**−**
7	**+**	**+**	**+**	**+**	**+**	**+**
8	**+**	**−**	**−**	**+**	**−**	**−**
9	**−**	**−**	**−**	**−**	**−**	**−**
11	**+**	**+**	**+**	**+**	**−**	**+**

P. murina cDNA	**+**	**+**	**+**	**+**	**+**	**+**

a +, positive PCR result; **−**, negative PCR result. The amplification results of the *P. jirovecii MAT* genes are from Richard et al. (17) (the BAL fluid sample from patient 10 could not be analyzed here because it was no longer available).

b Amplification of β-*tub* was used as control.

For a further control in these experiments, PCRs were designed to span an intron of the *mam2* and *map3* transcripts as for β*-tub*. The size of the PCR product from the *P. jirovecii mam2* transcripts revealed that intron 1 predicted in the Cissé assembly was not spliced (Fig. 2A). On the other hand, two PCR products were systematically obtained from the *map3* transcripts, one containing intron 1, the other not. For both genes, DNA sequencing of the PCR products of patients 1 and 5 confirmed the presence of the intron and revealed the absence of modification of its sequence relative to the *P. jirovecii* genome sequence. This suggested that (i) the absence of splicing of intron 1 of *mam2* was not due to polymorphisms, and (ii) the two *map3* transcripts resulted from alternative splicing, rather than from polymorphisms present in the different *P. jirovecii* strains that we found to coinfect all our samples.

We also investigated the expression of the two P. murina receptors as well as of the three *MAT* genes in one sample from the infected lungs of a single mouse. The sample was positive for the expression of all these genes, with splicing of the predicted intron 1 from both *mam2* and *map3* transcripts (Fig. 2B and Table 2).

In conclusion, our observations suggested that the two pheromone receptors are most often concomitantly expressed during *Pneumocystis* infection, both in humans and mice, and that the *P. jirovecii map3* transcripts are subject to alternative splicing.

### The *Pneumocystis* pheromone receptors Mam2 and Map3 localize within the cell membrane upon expression in S. cerevisiae.

**FIG 3:**
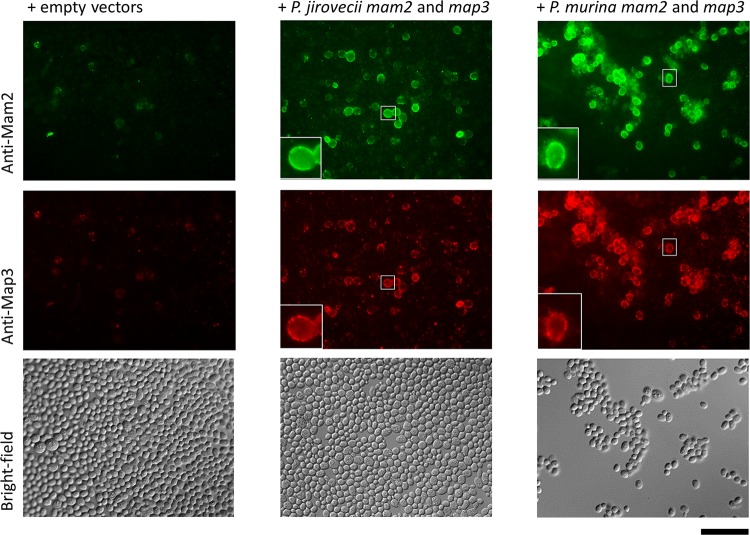
Indirect immunofluorescence microscopic analysis of *Pneumocystis* pheromone receptors Mam2 and Map3 expressed in S. cerevisiae. Recombinant SY2011 strains harbored plasmids expressing the indicated heterologous genes or empty vectors. Costaining anti-Mam2 and anti-Map3 was performed. A FITC filter was used to visualize Mam2 (green; Alexa Fluor 488), and a TRITC filter was used to visualize Map3 (red; Alexa Fluor 594). The cells in the small white boxes are shown enlarged in the insets in the bottom left corner of the image. Bar, 50 μm.

In order to assess their localization within the cell membrane, the *Pneumocystis* pheromone receptors were expressed in S. cerevisiae and visualized using specific antibodies directed against their extracellular domains (Fig. 1). These experiments were also meant to validate the staining tools needed to visualize the receptors at the surfaces of *Pneumocystis* cells from infected lungs. Because S. cerevisiae does not process *Pneumocystis* introns, synthetic *mam2* and *map3* genes without introns were cloned into plasmids for heterologous expression. The S. cerevisiae strain SY2011 used has both endogenous pheromone receptors deleted. Each recombinant strain expressing both Mam2 and Map3 of *P. jirovecii* or of P. murina was stained using two specific antibodies and two secondary antibodies of different fluorescent colors, and their cell surface was examined with a microscope. A strain carrying both empty vectors was used as a negative control. Strong green and red signals were observed at the surface of the vast majority of the cells expressing P. murina or *P. jirovecii* pheromone receptors, but not on the cells of the control containing the empty vectors (Fig. 3). Control experiments using strains harboring a single recombinant plasmid verified the specificity of each antibody (Fig. S4). Consistent with the expected localization of the receptors within the cellular membrane, the coloration appeared as little dots all around the cells at their surface, as shown by the cells enlarged in the insets in the bottom left corner of some images in Fig. 3. In these experiments, we used the *P. jirovecii* ORF alleles of the Cissé assembly, i.e., Mam2 without the 10 residues corresponding to the first intron, and Map3 missing 27 residues at the C terminus. These lacks did not affect membrane localization and recognition by the antibodies. In conclusion, these observations were consistent with a localization of both *Pneumocystis* Mam2 and Map3 pheromone receptors within the cellular membrane and validated the immunofluorescence staining tools.

10.1128/mBio.01145-19.4 FIG S4 Indirect immunofluorescence microscopic analysis of *Pneumocystis* pheromone receptors Mam2 and Map3 expressed in S. cerevisiae. Recombinant SY2011 strains harbored one or two plasmids expressing the indicated heterologous gene(s) from *P. jirovecii* (A) or P. murina (B), or empty vectors. Costaining with anti-Mam2 and anti-Map3 was performed. A FITC filter was used to visualize Mam2 (green; Alexa Fluor 488), and a TRITC filter was used to visualize Map3 (red; Alexa Fluor 594). Each scale bar is 50 μm. Download FIG S4, PDF file, 1.6 MB. Copyright © 2019 Luraschi et al. 2019 Luraschi et al. This is an open-access article distributed under the terms of the Creative Commons Attribution 4.0 International license.

### The Mam2 and Map3 receptors are frequently both present at the surfaces of *P. jirovecii* cells.

**FIG 4:**
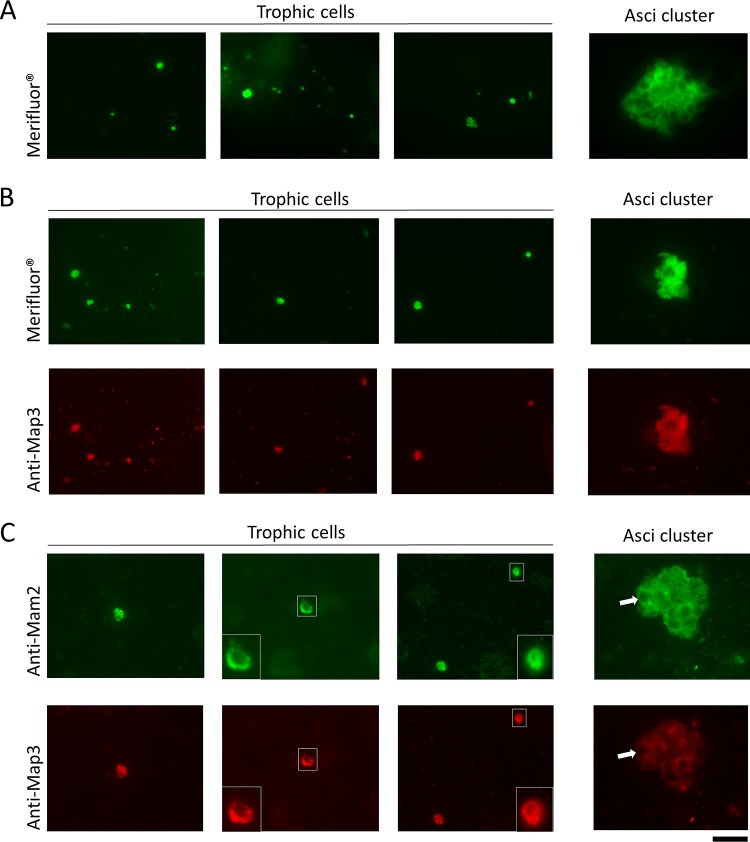
Indirect immunofluorescence microscopic analysis of Mam2 and Map3 pheromone receptors on *P. jirovecii* cells from a BAL fluid sample from a patient with PCP. (A) Merifluor staining. (B) Costaining Merifluor and anti-Map3. (C) Costaining anti-Mam2 and anti-Map3. The presumed trophic cells in the small white squares are shown enlarged in the insets at the left or right bottom corners of the images, and a single spherical cell corresponding to an ascus within the cluster is indicated by the white arrows. A FITC filter (green) was used to visualize Merifluor and Mam2 stainings (Alexa Fluor 488), and a TRITC filter (red) was used to visualize Map3 staining (Alexa Fluor 594). Bar, 25 μm.

The reverse transcriptase PCR analyses showed that both *mam2* and *map3* genes are often expressed concomitantly during *Pneumocystis* infection. This was compatible with two possibilities: each cell expresses only one of the two receptors, or each cell expresses both receptors. To investigate this issue, we performed indirect immunofluorescence stainings of the receptors on *P. jirovecii* cells from the BAL fluid sample of patient 1 (with the highest fungal load among our samples [17]). In order to differentiate trophic cells from asci, we used the Merifluor kit relying on antibodies directed against cell wall antigens of all *P. jirovecii* cell types (trophic forms, asci, and ascospores). This kit also stains the extracellular matrix surrounding the cells within clusters. Using this staining kit, the isolated cells are mostly trophic cells (2 to 8 μm), whereas the rounded cells within the clusters are mostly asci (4 to 6 μm; Fig. 4A and B, green stain). Thus, the isolated cells that are smaller than the asci seen in the clusters are most likely trophic forms. Consistent with targeting cell wall antigens, Merifluor stained the surface of the asci and the extracellular matrix within the clusters (Fig. 4A). Staining of the surfaces of the presumed trophic cells was not visualized, possibly because of intense staining. Costaining with Merifluor and anti-Map3 could be performed because of distinct fluorescent colors. It revealed that cells likely to be trophic forms were most often also positive with the Map3 staining (Fig. 4B). Costaining with anti-Mam2 and anti-Map3 antibodies revealed that the majority of presumed trophic cells were positive with both stainings (Fig. 4C). We observed 11 cells positive with the costaining with Merifluor and anti-Map3 and 13 cells positive with costaining with anti-Mam2 and anti-Map3; the most representative cells are shown in Fig. 4. Four or five cells were positive only with Merifluor, but not with anti-Map3 antibodies. Although we cannot exclude the possibility that the latter cells did not express Map3, they could also have resulted from the fading of immunofluorescence. The presumed trophic cells were often the most strongly stained (compare trophic cells and clusters of asci in Fig. 4B and C). This would be consistent with expression of the receptors mostly or exclusively by trophic cells. Staining within the clusters may have resulted from trophic cells that are also present in these structures. As expected, staining of the receptors was sometimes clearly located at the surfaces of the cells, as shown by those enlarged in Fig. 4C. In conclusion, these observations suggested that Mam2 and Map3 receptors are often both present at the surfaces of *P. jirovecii* trophic cells.

## DISCUSSION

The mode of sexual reproduction of *Pneumocystis* organisms is most probably primary homothallism. In the present study, we investigated the expression of the *Pneumocystis mam2* and *map3* genes that encode the receptors specific for the P and M mating pheromones. We found that both genes are most often expressed concomitantly during infection in humans as well as in mice. In addition, immunostainings revealed that both receptors are most often present at the surfaces of presumed trophic cells. Thus, *Pneumocystis* sexuality would involve cells that are of both mating types P and M at the same time. Being of both mating types, each trophic cell would be expected to excrete both pheromones P and M. We could not study this because the genes encoding these pheromones have not been identified (16, 19), which is consistent with their notoriously important divergence among fungi.

The *P. jirovecii mam2* transcripts contained an intron that was predicted in only one of the two genome assemblies. This intron might be an erroneous prediction because of the following. (i) It is the smallest *Pneumocystis* intron reported so far (30 bp versus the average of ca. 50 bp [9, 20–22]). (ii) Its translation product is present in other fungal orthologous receptors (Fig. 1). (iii) We show in the present study that it is not spliced during infection of humans. However, one cannot exclude the possibility that this intron is spliced in other conditions than those we investigated here. On the other hand, intron 1 was spliced in about half of the *P. jirovecii map3* transcripts, strongly suggesting a biological significance. This intron conserved the ORF and its translation product inserts within the fifth transmembrane domain (between positions 176 and 177 in Fig. 1). Although this translation product is not predicted to be part of the transmembrane domain (not shown), it is unknown whether its presence results in a functional protein. Our observations for *map3* transcripts can be attributed to the occurrence of alternative splicing events, a mechanism that has already been reported for the *map3* transcripts of P. carinii (23). Alternative splicing is thought to be used to increase the diversity of transcripts, possibly to respond to different environments, as well as to regulate gene transcription (24). Intron retention is thought to be involved in the regulation of protein isoform production. A high level of alternative splicing events has been reported in *Pneumocystis* species, with intron retention being the most common mechanism (42% to 49% of the introns concerned [19]). The different splicing variants could be associated with the different *Pneumocystis* cellular forms (25) or with the asexual and sexual cycles, as reported in Aspergillus nidulans (26). Interestingly, the intron retained in half of the *map3* transcripts was predicted only in the Cissé assembly, not in the Ma assembly. This difference might result from differences between the prediction tools used. As far as P. murina is concerned, the predicted introns were spliced from the *mam2* and *map3* transcripts. These two introns contain a stop codon in the frame of the ORF, suggesting that the nonsense-mediated mRNA decay machinery, a pathway existing in all eukaryotes, including *Pneumocystis* (19), eliminates transcripts retaining these introns.

Primary homothallism is hypothesized to be advantageous for pathogenic fungi because it avoids the need to find a compatible mating partner, while still providing evolutionary advantages (27). Indeed, even if it involves a single strain, primary homothallism can avoid accumulating deleterious mutations and increase genetic diversity as well as virulence (28). The mechanisms involved in this mode of sexual reproduction remain poorly characterized compared to those in heterothallic and secondary homothallic fungi. Given that each strain is self-fertile, the pheromone-receptor systems might be dispensable because recognition of the mating partner is not needed. However, they might be involved in postfertilization processes, such as recognition of the nuclei during the formation of dikaryotic cells (26). For example, the plant pathogen *Taphrina deformans* does not rely on cell-cell fusion in order to enter into its homothallic sexual cycle (29, 30), but it possesses a potential Map3 receptor (16). Like *Pneumocystis*, other primary homothallic fungi also express two pheromone-receptor systems, e.g., A. nidulans (26), Phaffia rhodozyma (31), Sordaria macrospora (32), *Neurospora* species (33), and Gibberella zeae (34). Both systems are necessary for sexuality to occur in A. nidulans, whereas only one is required in S. macrospora and P. rhodozyma, and none in G. zeae. Thus, the pheromone-receptor systems might also have nonessential functions in sexuality. As far as *Pneumocystis* species are concerned, electron microscope studies suggested that cell-cell fusion may occur (7), so that the two pheromone-receptor systems might be involved at that stage. In the case of *P. jirovecii*, the presence of pheromone receptors at the cell surface might facilitate outbreeding through mating with the other strains that are most often, if not always, present in each human infection (35). Such outbreeding might allow increasing the genetic diversity compared to inbreeding through self-fertilization of a single strain. The situation might be different in P. murina infections because these are apparently monoclonal (36; M. T. Cushion, unpublished data), although the situation in wild animals remains unclear.

In the closely related organism S. pombe, the expression of the Map3 pheromone receptor is regulated by the MatPc transcription factor. This could not be the case in *Pneumocystis* organisms because no MatPc homolog was identified (16), suggesting that another transcription factor regulates the genes specific to mating type P. In S. pombe, the fusion between two cells of opposite mating type allows the formation of a complex composed of Pi and Mi transcription factors, Pi originating from the P cell, Mi from the M cell (37, 38). This complex activates *mei3*, a gene involved in meiosis induction and inhibition of refertilization. The mechanisms involved in *Pneumocystis* organisms are different because (i) the *mei3* gene seems not to be present in *Pneumocystis* genomes (16), and (ii) the Mi and Pi transcription factors are potentially expressed in each cell, so that the Pi-Mi complex might be permanently formed. Thus, the molecular mechanisms involved in *Pneumocystis* sexuality are different from those in S. pombe. This conclusion is not surprising because rewiring of *MAT* pathways is a phenomenon frequently observed among fungi (39, 40).

In conclusion, our results suggest that both Mam2 and Map3 pheromone receptors are most often both present on *P. jirovecii* trophic cells. Thus, each cell would be of both M and P mating types at the same time. The presence of receptors at the cell surface might facilitate outbreeding versus inbreeding of self-fertile strains. Further endeavors are necessary to decipher the function(s) of the two pheromone-receptor systems in *Pneumocystis* sexuality.

## MATERIALS AND METHODS

### Strain and growth conditions.

SY2011 is an S. cerevisiae haploid strain in which both mating receptors *STE2* and *STE3* are deleted (*MAT****a*** *ste3Δ ste2Δ mfa1Δ mfa2*Δ::*FUS1-LacZ*) (41). It was grown at 30°C on complete yeast extract-peptone-dextrose (YEPD) medium (1% [wt/vol] Difco yeast extract, 2% Difco peptone, 2% glucose). SY2011 grows as pale pink colonies because it carries mutations within the adenine biosynthesis pathway.

### Source and cloning of the *Pneumocystis mam2* and *map3* genes.

To identify the P. murina and *P. jirovecii mam2* genes encoding the receptor for P factor, the Mam2 protein of P. carinii (UniProt ID A2TJ26) was used as the query sequence in BLASTp searches at http://blast.ncbi.nlm.nih.gov/Blast.cgi against all proteomes of the genus *Pneumocystis*. A single putative ortholog was detected in each of the two *P. jirovecii* proteomes available: PNEJI1_000211 locus in the assembly version ASM33397v2 (9; the Cissé assembly), and T551_00015 locus in the assembly version Pneu-jiro_RU7_V2 (19; the Ma assembly). A single putative *mam2* ortholog was also found in P. murina (PNEG_03148). The P. murina and *P. jirovecii* gene sequences encoding the Mam2 proteins were then retrieved from the European Nucleotide Archive (http://www.ebi.ac.uk/ena). The same procedure was applied to retrieve the P. murina and *P. jirovecii map3* genes encoding the M receptor using the P. carinii Map3 (Q9HDG3). A single putative ortholog was detected in each *P. jirovecii* proteome: PNEJI1_002694 locus in the Cissé assembly and T551_02750 locus in the Ma assembly. A single putative *map3* ortholog was found in P. murina (PNEG_03013). The same genes were identified using Mam2 (Q00619) and Map3 (P31397) proteins of the well-annotated Taphrinomycotina member S. pombe as the query sequences. The *mam2* and *map3* genes we identified here corresponded to the candidates previously reported (16, 19). Glycosylphosphatidylinositol anchor signals were searched using GPI-SOM (http://gpi.unibe.ch) (42). Canonical TATA box and cap signal (43), as well as canonical donor, acceptor, as well as branch site and point sequences of *Pneumocystis* introns (20, 21), were identified by visual inspection of the alignments and sequences of the genes. Multiple sequence alignments of proteins were generated using T-Coffee (44). Sequence identity (as a percentage) of whole proteins has been calculated with Align Sequence Protein BLAST tool (http://blast.ncbi.nlm.nih.gov/Blast.cgi). Transmembrane domains and other conserved domains were identified using the NCBI’s search tool (https://www.ncbi.nlm.nih.gov/Structure/cdd/wrpsb.cgi). The extracellular domains, intracellular domains, and cytoplasmic C-terminal regions correspond to those predicted for the S. cerevisiae *Ste2* and *Ste3* (45), as well as P. carinii Map3 receptors (18). The *P. jirovecii* ORF alleles of the Cissé assembly were investigated in the present study. Because of the presence of two introns in each *mam2* or *map3* ORF, their cDNAs were synthesized with codon usage optimized for S. cerevisiae by GeneCust Europe (Ellange, Luxembourg). This step was necessary because S. cerevisiae does not process *Pneumocystis* introns. The *mam2* genes were cloned into p415GPD, while the *map3* genes were cloned into p416GPD (both plasmids from reference 46). Two plasmids with different markers were used so that they could be introduced at the same time in the S. cerevisiae strain SY2011 and selected for by leucine (p415GPD) and uracil (p416GPD) prototrophy.

### Source of the P. murina *mat* genes.

The P. murina *matMc* and *matPi* genes were identified using the *P. jirovecii* MatMc (locus T551_0262) and MatPi (T551_02159) proteins as query sequences in a BLASTp search against the P. murina proteome. A single putative ortholog of each Mat protein was detected: PNEG_02275 (MatMc), PNEG_02273 (MatPi). Because it is highly divergent, the P. murina *matMi* was identified as described by Almeida et al. (16) (GenBank accession no. AFWA02000013: coordinates 80789 to 80905).

### PCR amplification.

In order to avoid contaminations, PCRs were set up and analyzed in separate rooms, and negative controls were systematically performed at each experiment. All PCRs were performed using the High Fidelity Expand polymerase (Roche). The final concentrations of MgCl_2_ were 4.5 and 3 mM for all *P. jirovecii* and P. murina genes, respectively. Each reaction began with denaturation for 3 min at 94°C, followed by 35 cycles, with 1 cycle consisting of 30 s at 94°C, 30 s at the annealing temperature given in Table S1 in the supplemental material, and 30 s of elongation at 72°C. The reactions ended with an extension step of 10 min at 72°C. PCRs were performed on genomic DNA or cDNA obtained from BAL fluid samples from patients with *Pneumocystis* pneumonia (we already investigated these samples for the expression of *MAT* genes [17]). All these samples were found to be coinfected using the procedure previously described (47). Genomic DNAs were extracted using the QIAamp DNA blood kit (Qiagen). Total RNAs were extracted from the BAL fluid samples using the RiboPure yeast kit (Ambion). The clinical samples were previously frozen and stored at −80°C in RNA*later* (Ambion) as quickly as possible upon reception. cDNAs were synthesized from each RNA preparation using the REPLI-g WTA Single Cell kit involving random amplification (Qiagen). The resulting cDNA were purified using LiCl-ethanol precipitation in the presence of glycogen (Qiagen supplementary protocol). The random amplification of cDNA included in the kit was necessary for the detection of *P. jirovecii* transcripts among those of the patient because of their small amount. For the amplification of P. murina pheromone receptor genes, DNA and cDNA were obtained using the immunosuppressed mouse model of pneumocystosis previously described (48). Briefly, C3H/HeN mice (Charles River Laboratories) were exposed to infected mice for 2 weeks and started an immunosuppressive regimen the first day of exposure (4 μg/ml dexamethasone in drinking water). Mice were sacrificed 5 weeks after the initial exposure to P. murina, and their lungs were resected. Genomic DNA was extracted from lung homogenate from a pool of 10 infected immunosuppressed mice using the Blood and Tissue kit (Qiagen). Total RNA was extracted from a single mouse using TRIzol reagent (Thermo Fisher), and cDNAs were synthesized using the SuperScript IV Vilo Master Mix kit (Invitrogen). PCRs were designed to overlap one intron in order to show its splicing and assess the absence of genomic DNA. The primers used and conditions for amplification are listed in Table S1. Primers were synthesized by Microsynth (Balgach, Switzerland). Sequencing of both strands of the PCR products was performed with the two primers used for PCR amplification, as well as the BigDye Terminator DNA sequencing kit and ABI PRISM 3100 automated sequencer (both from PerkinElmer Biosystems).

10.1128/mBio.01145-19.5 TABLE S1 PCR primers and conditions. Download Table S1, DOCX file, 0.02 MB. Copyright © 2019 Luraschi et al. 2019 Luraschi et al. This is an open-access article distributed under the terms of the Creative Commons Attribution 4.0 International license.

### Transformation of the S. cerevisiae *ste2 ste3* double deletion strain.

The recombinant plasmids p415GPD containing the *P. jirovecii mam2* gene and p416GPD containing the *P. jirovecii map3* gene were cointroduced into the S. cerevisiae strain SY2011 by transformation for uracil and leucine prototrophy using the one-step method (49). The same was done using the recombinant plasmids containing the P. murina *mam2* and *map3* genes. For a control, strain SY2011 was cotransformed with empty p415GPD and p416GPD plasmids. Strains harboring only one of the four recombinant plasmids were also constructed in order to assess the specificity of each antibody. Transformants were selected on solid yeast nitrogen base (YNB) medium (0.67% [wt/vol] yeast nitrogen base, 2% glucose, 2% Gibco agar) supplemented with a complete supplement mixture lacking uracil and/or leucine (CSM) (MP Biomedicals).

### Antibody preparation.

Two peptides of the first extracellular domain of *P. jirovecii* Mam2 were selected: C+TVNQTVILKNSHGEK and C+LSDFDMFSLSRAQ (peptides P1 [peptide 1] and P2 [Fig. 1A]). Polyclonal antibodies against these peptides were prepared by Eurogentec by immunization of two rabbits, followed by mixing the sera obtained after 1 month for the staining experiments. Antibodies against two extracellular domains of *P. jirovecii* Map3 were similarly prepared by immunizing rats with the following peptides: WFNGVEAKSPGYIYC and QSKINYSWDHVHQWG+C (peptides P1 and P2 [Fig. 1B]). The same procedure was used for the preparation of the antibodies against P. murina Mam2 in rabbits using C+PTNQTIFLKNSKGET and LSDFDEFSLSRAQ+C peptides and against P. murina Map3 in rats using peptides WFNGSKSTYRGDLYC and KSNIKYSWSDVHNWN+C. These peptides are localized at the same positions as those for *P. jirovecii* (Fig. 1). Goat anti-rabbit IgG (H+L) highly cross-adsorbed secondary antibody Alexa Fluor Plus 488 (green; Invitrogen), and goat anti-Rat IgG (H+L) Alexa Fluor 594 (red; Eurogentec) were used to detect the primary antibodies.

### Indirect immunofluorescence staining and microscopy.

In order to avoid the loss of the plasmids, the S. cerevisiae strains expressing the *Pneumocystis* pheromone receptors were grown overnight in selective minimal medium YNB lacking uracil and/or leucine. Cells in stationary phase were diluted and grown from an optical density at 540 nm of 0.5 to 1.0 with shaking at 30°C in the same medium (ca. 7.5 × 10^6^ cells). One milliliter was then centrifuged for 5 min at 5,000 rpm and washed once with 1 ml phosphate-buffered saline (PBS) (0.68% NaCl, 0.04% KH_2_PO_4_, 0.15% Na_2_HPO_4_). Cells were resuspended in 50 μl nuclease-free water, and 15 μl was deposited on a microscope slide, which had been previously washed with 100% ethanol (EtOH), and dried. Once completely air dried at room temperature, cells were heat fixed by repeated rapid passages of the back side of the slide over a flame. Cells fixed on slides were treated with 100 μl blocking buffer (PBS with 5% normal goat serum [Gibco Life Technologies] and 3% bovine serum albumin [SERVA] [PBS/NGS 5%/BSA 3%) for 30 min at room temperature. Slides were then washed and covered with a mix of the anti-Mam2 and anti-Map3 primary antibodies with a final dilution of 1/25 in washing buffer (PBS/NGS 5%/BSA 0.01%). The slides were then incubated for 1 h at room temperature in a humid chamber. The humid chamber consisted of a plastic box containing wet absorbent paper with plastic supports on which slides were leaned. Antibodies were removed by washing the slide several times with washing buffer. Subsequently, slides were treated with a mix of two secondary antibodies (anti-rabbit and anti-rat) at a final dilution of 1/200 in washing buffer and incubated for 1 h at room temperature in the humid chamber. Secondary antibodies were removed, and the slides were washed several times with PBS. Slides mounted in water were then observed with a fluorescence microscope (Axioplan 2; Zeiss). To visualize the *P. jirovecii* cells in the BAL fluid samples from patients, the Merilfluor coloration (Meridian, Bioscience Europe) was used according to the manufacturer’s instructions. A fluorescein isothiocyanate (FITC) filter was used to visualize Mam2 receptors and Merifluor staining (green fluorescence), whereas a tetramethylrhodamine isothiocyanate (TRITC) filter was used to visualize Map3 receptor (red fluorescence). All pictures were taken at ×1,000 magnification, with a Spot RT3 camera (Visitron System). ImageJ was used to handle the images.
