# Supplementary material for: Expression and Immunostaining Analyses Suggest that Pneumocystis Primary Homothallism Involves Trophic Cells Displaying Both Plus and Minus Pheromone Receptors
Source: mBio. 2019 Jul 9;10(4):e01145-19. doi: 10.1128/mBio.01145-19 (PMC6747714; doi:10.1128/mBio.01145-19)
Supplement: FIG S1 [file mBio.01145-19-sf001.docx]

**Fig. S1**

**A**

Pjmam2_Cissé_ORF 1 ATGTCTCTTTCTACAGTAAACCAGACTGTCATTCTTAAGAATTCTCATGGAGAGAAGGTCAAATTT 66

Pjmam2_Cissé_gen 1 ATGTCTCTTTCTACAGTAAACCAGACTGTCATTCTTAAGAATTCTCATGGAGAGAAGGTCAAATTT 66

Pjmam2_Ma_ORF 1 ATGTCTCTTTCTACAGTAAACCAGACTGTCATTCTTAAGAATTCTCATGGAGAGAAGGTCAAATTT 66

Pjmam2_Ma_gen 1 ATGTCTCTTTCTACAGTAAACCAGACTGTCATTCTTAAGAATTCTCATGGAGAGAAGGTCAAATTT 66

1 ****************************************************************** 66

Pjmam2_Cissé_ORF 67 TTATTGTCAGATTTTGACATGTTTTCTCTTTCTAGAGCACAAACATCCATGATATTTTCTGCACAA 132

Pjmam2_Cissé_gen 67 TTATTGTCAGATTTTGACATGTTTTCTCTTTCTAGAGCACAAACATCCATGATATTTTCTGCACAA 132

Pjmam2_Ma_ORF 67 TTATTGTCAGATTTTGACATGTTTTCTCTTTCTAGAGCACAAACATCCATGATATTTTCTGCACAA 132

Pjmam2_Ma_gen 67 TTATTGTCAGATTTTGACATGTTTTCTCTTTCTAGAGCACAAACATCCATGATATTTTCTGCACAA 132

67 ****************************************************************** 132

Pjmam2_Cissé_ORF 133 TGTGCAATGAGTGCTTTGTTAGCAATTATTCTTCTATTAACATCAAAACGTGAAAAAGCAAAAACA 198

Pjmam2_Cissé_gen 133 TGTGCAATGAGTGCTTTGTTAGCAATTATTCTTCTATTAACATCAAAACGTGAAAAAGCAAAAACA 198

Pjmam2_Ma_ORF 133 TGTGCAATGAGTGCTTTGTTAGCAATTATTCTTCTATTAACATCAAAACGTGAAAAAGCAAAAACA 198

Pjmam2_Ma_gen 133 TGTGCAATGAGTGCTTTGTTAGCAATTATTCTTCTATTAACATCAAAACGTGAAAAAGCAAAAACA 198

133 ****************************************************************** 198

Pjmam2_Cissé_ORF 199 TTTCTTTTCTTTTTAAACATGGCTGGACTAATATCTGTATTTATACGAGGATGCCTTCAATGTGCT 264

Pjmam2_Cissé_gen 199 TTTCTTTTCTTTTTAAACATGGCTGGACTAATATCTGTATTTATACGAGGATGCCTTCAATGTGCT 264

Pjmam2_Ma_ORF 199 TTTCTTTTCTTTTTAAACATGGCTGGACTAATATCTGTATTTATACGAGGATGCCTTCAATGTGCT 264

Pjmam2_Ma_gen 199 TTTCTTTTCTTTTTAAACATGGCTGGACTAATATCTGTATTTATACGAGGATGCCTTCAATGTGCT 264

199 ****************************************************************** 264

Pjmam2_Cissé_ORF 265 TATTTAACTGGTACATGGACAAGCTATAGTGTTCAATTTCTCGGAGAATTCGAGTTGTTATCATAT 330

Pjmam2_Cissé_gen 265 TATTTAACTGGTACATGGACAAGCTATAGTGTTCAATTTCTCGGAGAATTCGAGTTGTTATCATAT 330

Pjmam2_Ma_ORF 265 TATTTAACTGGTACATGGACAAGCTATAGTGTTCAATTTCTCGGAGAATTCGAGTTGTTATCATAT 330

Pjmam2_Ma_gen 265 TATTTAACTGGTACATGGACAAGCTATAGTGTTCAATTTCTCGGAGAATTCGAGTTGTTATCATAT 330

265 ****************************************************************** 330

Pjmam2_Cissé_ORF 331 AATGATTTCTATGTCTCAATTATTGCATCATGCATGCCTATTTTTATCATCTTATTTATTGAGCTT 396

Pjmam2_Cissé_gen 331 AATGATTTCTATGTCTCAATTATTGCATCATGCATGCCTATTTTTATCATCTTATTTATTGAGCTT 396

Pjmam2_Ma_ORF 331 AATGATTTCTATGTCTCAATTATTGCATCATGCATGCCTATTTTTATCATCTTATTTATTGAGCTT 396

Pjmam2_Ma_gen 331 AATGATTTCTATGTCTCAATTATTGCATCATGCATGCCTATTTTTATCATCTTATTTATTGAGCTT 396

331 ****************************************************************** 396

Pjmam2_Cissé_ORF 397 TCTCTTCTTATTCAAATTAGAGTAATCTACGCATCACACAGAAAGTTACGAATGCCGCTCACAATA 462

Pjmam2_Cissé_gen 397 TCTCTTCTTATTCAAATTAGAGTAATCTACGCATCACACAGAAAGTTACGAATGCCGCTCACAATA 462

Pjmam2_Ma_ORF 397 TCTCTTCTTATTCAAATTAGAGTAATCTACGCATCACACAGAAAGTTACGAATGCCGCTCACAATA 462

Pjmam2_Ma_gen 397 TCTCTTCTTATTCAAATTAGAGTAATCTACGCATCACACAGAAAGTTACGAATGCCGCTCACAATA 462

397 ****************************************************************** 462

Pjmam2_Cissé_ORF 463 ATTTCTTGTGTCATAATATCAGTAGTTATACTATTTTGGGTAATTGCTGCTATTCAAAATTCAATG 528

Pjmam2_Cissé_gen 463 ATTTCTTGTGTCATAATATCAGTAGTTATACTATTTTGGGTAATTGCTGCTATTCAAAATTCAATG 528

Pjmam2_Ma_ORF 463 ATTTCTTGTGTCATAATATCAGTAGTTATACTATTTTGGGTAATTGCTGCTATTCAAAATTCAATG 528

Pjmam2_Ma_gen 463 ATTTCTTGTGTCATAATATCAGTAGTTATACTATTTTGGGTAATTGCTGCTATTCAAAATTCAATG 528

463 ****************************************************************** 528

Pjmam2_Cissé_ORF 529 GCGATTTTGTCTCAAACACATTTTGGAAGCAGTGGTATTTGGGGTGCACCTTGGCCATATACCGCC 594

Pjmam2_Cissé_gen 529 GCGATTTTGTCTCAAACACATTTTGGAAGCAGTGGTATTTGGGGTGCACCTTGGCCATATACCGCC 594

Pjmam2_Ma_ORF 529 GCGATTTTGTCTCAAACACATTTTGGAAGCAGTGGTATTTGGGGTGCACCTTGGCCATATACCGCC 594

Pjmam2_Ma_gen 529 GCGATTTTGTCTCAAACACATTTTGGAAGCAGTGGTATTTGGGGTGCACCTTGGCCATATACCGCC 594

529 ****************************************************************** 594

___________intron 1___________

Pjmam2_Cissé_ORF 595 GCCC------------------------------GATGTTTAGTTTTTGTTTCAAAATTATTTTTC 630

Pjmam2_Cissé_gen 595 GCCC**GT**ATTTCATTTGTTT**T**T**A**GTGTATGTTT**AG**GATGTTTAGTTTTTGTTTCAAAATTATTTTTC 660

Pjmam2_Ma_ORF 595 GCCC**GT**ATTTCATTTGTTT**T**T**A**GTGTATGTTT**AG**GATGTTTAGTTTTTGTTTCAAAATTATTTTTC 660

Pjmam2_Ma_gen 595 GCCC**GT**ATTTCATTTGTTT**T**T**A**GTGTATGTTT**AG**GATGTTTAGTTTTTGTTTCAAAATTATTTTTC 660

595 **** ******************************** 660

Pjmam2_Cissé_ORF 631 GCAATTTACCGTAGACATAAAATGGGTATCAAAGATTTTGGACCAATGCAAATCATATTTATTACA 696

Pjmam2_Cissé_gen 661 GCAATTTACCGTAGACATAAAATGGGTATCAAAGATTTTGGACCAATGCAAATCATATTTATTACA 726

Pjmam2_Ma_ORF 661 GCAATTTACCGTAGACATAAAATGGGTATCAAAGATTTTGGACCAATGCAAATCATATTTATTACA 726

Pjmam2_Ma_gen 661 GCAATTTACCGTAGACATAAAATGGGTATCAAAGATTTTGGACCAATGCAAATCATATTTATTACA 726

661 ****************************************************************** 726

_________________________intron 2________

Pjmam2_Cissé_ORF 697 AGCTGTCAAACATTAATTATTCCTG----------------------------------------- 720

Pjmam2_Cissé_gen 727 AGCTGTCAAACATTAATTATTCCTG**GT**ATCTTTTTTTTTCTTTTTTTCCCTTTACTCT**T**A**A**CACTT 792

Pjmam2_Ma_ORF 727 AGCTGTCAAACATTAATTATTCCTG----------------------------------------- 750

Pjmam2_Ma_gen 727 AGCTGTCAAACATTAATTATTCCTG**GT**ATCTTTTTTTTTCTTTTTTTCCCTTTACTCT**T**A**A**CACTT 792

727 ************************* 792

____

Pjmam2_Cissé_ORF 721 ----CAATCTTTATTATTATTGATTTCTGGGTAGATATAACCGGATTTAGTTCATTGACTCAAGCG 783

Pjmam2_Cissé_gen 793 TT**AG**CAATCTTTATTATTATTGATTTCTGGGTAGATATAACCGGATTTAGTTCATTGACTCAAGCG 858

Pjmam2_Ma_ORF 751 ----CAATCTTTATTATTATTGATTTCTGGGTAGATATAACCGGATTTAGTTCATTGACTCAAGCG 813

Pjmam2_Ma_gen 793 TT**AG**CAATCTTTATTATTATTGATTTCTGGGTAGATATAACCGGATTTAGTTCATTGACTCAAGCG 858

793 ************************************************************** 858

Pjmam2_Cissé_ORF 784 TTTGTTGTAATGTCTTTACCATTATCTTCTCTTTGGGCATCATCTAAAATAGAAAAAAATAAAAAT 849

Pjmam2_Cissé_gen 859 TTTGTTGTAATGTCTTTACCATTATCTTCTCTTTGGGCATCATCTAAAATAGAAAAAAATAAAAAT 924

Pjmam2_Ma_ORF 814 TTTGTTGTAATGTCTTTACCATTATCTTCTCTTTGGGCATCATCTAAAATAGAAAAAAATAAAAAT 879

Pjmam2_Ma_gen 859 TTTGTTGTAATGTCTTTACCATTATCTTCTCTTTGGGCATCATCTAAAATAGAAAAAAATAAAAAT 924

859 ****************************************************************** 924

Pjmam2_Cissé_ORF 850 AGCATGGCACAGCCATACAGTGAGCGTATAAATAGCAAGGATTATAGTGTTAAAAGCTCTCCAACT 915

Pjmam2_Cissé_gen 925 AGCATGGCACAGCCATACAGTGAGCGTATAAATAGCAAGGATTATAGTGTTAAAAGCTCTCCAACT 990

Pjmam2_Ma_ORF 880 AGCATGGCACAGCCATACAGTGAGCGTATAAATAGCAAGGATTATAGTGTTAAAAGCTCTCCAACT 945

Pjmam2_Ma_gen 925 AGCATGGCACAGCCATACAGTGAGCGTATAAATAGCAAGGATTATAGTGTTAAAAGCTCTCCAACT 990

925 ****************************************************************** 990

Pjmam2_Cissé_ORF 916 TCGCTAAGTAAATCATCTTATATCGACTTTAAACAGCCCCCTTGTTATTTAGATTCTGGGAAATCA 981

Pjmam2_Cissé_gen 991 TCGCTAAGTAAATCATCTTATATCGACTTTAAACAGCCCCCTTGTTATTTAGATTCTGGGAAATCA 1056

Pjmam2_Ma_ORF 946 TCGCTAAGTAAATCATCTTATATCGACTTTAAACAGCCCCCTTGTTATTTAGATTCTGGGAAATCA 1011

Pjmam2_Ma_gen 991 TCGCTAAGTAAATCATCTTATATCGACTTTAAACAGCCCCCTTGTTATTTAGATTCTGGGAAATCA 1056

991 ****************************************************************** 1056

Pjmam2_Cissé_ORF 982 CCATGTATTCCTTCTTTTGAATATAATGGAAATCCTTTTGATCAATTTTATGAAAATGACAGAAAT 1047

Pjmam2_Cissé_gen 1057 CCATGTATTCCTTCTTTTGAATATAATGGAAATCCTTTTGATCAATTTTATGAAAATGACAGAAAT 1122

Pjmam2_Ma_ORF 1012 CCATGTATTCCTTCTTTTGAATATAATGGAAATCCTTTTGATCAATTTTATGAAAATGACAGAAAT 1077

Pjmam2_Ma_gen 1057 CCATGTATTCCTTCTTTTGAATATAATGGAAATCCTTTTGATCAATTTTATGAAAATGACAGAAAT 1122

1057 ****************************************************************** 1122

Pjmam2_Cissé_ORF 1048 AGACTCAATATATTTATAGAAGAATCAGTAGATATATCTTCAGAAAAAGCATAA 1101

Pjmam2_Cissé_gen 1123 AGACTCAATATATTTATAGAAGAATCAGTAGATATATCTTCAGAAAAAGCATAA 1176

Pjmam2_Ma_ORF 1078 AGACTCAATATATTTATAGAAGAATCAGTAGATATATCTTCAGAAAAAGCATAA 1131

Pjmam2_Ma_gen 1123 AGACTCAATATATTTATAGAAGAATCAGTAGATATATCTTCAGAAAAAGCATAA 1176

1123 ****************************************************** 1176

**B**

Pjmap3_Cissé_ORF 1 ----------------------------------------------------------------- 2

Pjmap3_Cissé_gen 1 ----------------------------------------------------------------- 2

Pjmap3_Ma_ORF 1 ATGTATTTGGGTTAACGGAGCATATAAGTTGTTTTCAAGTATTACGTGTCCAATTTTTTGTTGAT 66

Pjmap3_Ma_gen 1 ATGTATTTGGGTTAACGGAGCATATAAGTTGTTTTCAAGTATTACGTGTCCAATTTTTTGTTGAT 66

1 66

Pjmap3_Cissé_ORF 3 ----ATGGGCGATGTATTTTACGTTATTTATTCTTTTATTGGATTTATATGCTCAGTTATACCTTC 62

Pjmap3_Cissé_gen 3 ----ATGGGCGATGTATTTTACGTTATTTATTCTTTTATTGGATTTATATGCTCAGTTATACCTTC 62

Pjmap3_Ma_ORF 67 CAAGATGGGCGATGTATTTTACGTTATTTATTCTTTTATTGGATTTATATGCTCAGTTATACCTTC 132

Pjmap3_Ma_gen 67 CAAGATGGGCGATGTATTTTACGTTATTTATTCTTTTATTGGATTTATATGCTCAGTTATACCTTC 132

67 ****************************************************************** 132

Pjmap3_Cissé_ORF 63 TATTTGGCACTGGAAATATCGTAATGTAGCACCACTATGTCTTATTTTTTGGATCTCTGCTTGCAG 128

Pjmap3_Cissé_gen 63 TATTTGGCACTGGAAATATCGTAATGTAGCACCACTATGTCTTATTTTTTGGATCTCTGCTTGCAG 128

Pjmap3_Ma_ORF 133 TATTTGGCACTGGAAATATCGTAATGTAGCACCACTATGTCTTATTTTTTGGATCTCTGCTTGCAG 198

Pjmap3_Ma_gen 133 TATTTGGCACTGGAAATATCGTAATGTAGCACCACTATGTCTTATTTTTTGGATCTCTGCTTGCAG 198

133 ****************************************************************** 198

Pjmap3_Cissé_ORF 129 CTTAATATGTTTTATAAACTCTATCGTCTGGTTTAATGGAGTTGAAGCAAAATCTCCGGGTTATAT 194

Pjmap3_Cissé_gen 129 CTTAATATGTTTTATAAACTCTATCGTCTGGTTTAATGGAGTTGAAGCAAAATCTCCGGGTTATAT 194

Pjmap3_Ma_ORF 199 CTTAATATGTTTTATAAACTCTATCGTCTGGTTTAATGGAGTTGAAGCAAAATCTCCGGGTTATAT 264

Pjmap3_Ma_gen 199 CTTAATATGTTTTATAAACTCTATCGTCTGGTTTAATGGAGTTGAAGCAAAATCTCCGGGTTATAT 264

199 ****************************************************************** 264

Pjmap3_Cissé_ORF 195 ATACTG**T**GATATTGCTACAAAAATTATACTTGGGTCTACCTCAGGAGAATTAGGTGCTATTGCAGC 260

Pjmap3_Cissé_gen 195 ATACTG**T**GATATTGCTACAAAAATTATACTTGGGTCTACCTCAGGAGAATTAGGTGCTATTGCAGC 260

Pjmap3_Ma_ORF 265 ATACTG**C**GATATTGCTACAAAAATTATACTTGGGTCTACCTCAGGAGAATTAGGTGCTATTGCAGC 330

Pjmap3_Ma_gen 265 ATACTG**C**GATATTGCTACAAAAATTATACTTGGGTCTACCTCAGGAGAATTAGGTGCTATTGCAGC 330

265 ****** *********************************************************** 330

Pjmap3_Cissé_ORF 261 TATTTCACATTATCTTTCAAAAATCATGAGCCCTGTACATTCTTCTGTACAAACTAAAACAAT**A**CG 326

Pjmap3_Cissé_gen 261 TATTTCACATTATCTTTCAAAAATCATGAGCCCTGTACATTCTTCTGTACAAACTAAAACAAT**A**CG 326

Pjmap3_Ma_ORF 331 TATTTCACATTATCTTTCAAAAATCATGAGCCCTGTACATTCTTCTGTACAAACTAAAACAAT**C**CG 396

Pjmap3_Ma_gen 331 TATTTCACATTATCTTTCAAAAATCATGAGCCCTGTACATTCTTCTGTACAAACTAAAACAAT**C**CG 396

331 *************************************************************** ** 396

Pjmap3_Cissé_ORF 327 CAGAAGACAGGCTATAGAAGATCTTCTTATGAGTTTTACATGCCCAATCATCATGATATGTTTA**C**A 392

Pjmap3_Cissé_gen 327 CAGAAGACAGGCTATAGAAGATCTTCTTATGAGTTTTACATGCCCAATCATCATGATATGTTTA**C**A 392

Pjmap3_Ma_ORF 397 CAGAAGACAGGCTATAGAAGATCTTCTTATGAGTTTTACATGCCCAATCATCATGATATGTTTA**T**A 462

Pjmap3_Ma_gen 397 CAGAAGACAGGCTATAGAAGATCTTCTTATGAGTTTTACATGCCCAATCATCATGATATGTTTA**T**A 462

397 **************************************************************** * 462

Pjmap3_Cissé_ORF 393 TTATGTTATTCAATCTGCAAGGTATGTAATAAATGGTGTCAATGGGTGTGTACCATGGTCCGATCA 458

Pjmap3_Cissé_gen 393 TTATGTTATTCAATCTGCAAGGTATGTAATAAATGGTGTCAATGGGTGTGTACCATGGTCCGATCA 458

Pjmap3_Ma_ORF 463 TTATGTTATTCAATCTGCAAGGTATGTAATAAATGGTGTCAATGGGTGTGTACCATGGTCCGATCA 528

Pjmap3_Ma_gen 463 TTATGTTATTCAATCTGCAAGGTATGTAATAAATGGTGTCAATGGGTGTGTACCATGGTCCGATCA 528

463 ****************************************************************** 528

Pjmap3_Cissé_ORF 459 ATCATGGCCAACAGTAATCATCGTTTTAATCTGGCCCCCTATTTTTGGTTCAATCAGTGCTTATTA 524

Pjmap3_Cissé_gen 459 ATCATGGCCAACAGTAATCATCGTTTTAATCTGGCCCCCTATTTTTGGTTCAATCAGTGCTTATTA 524

Pjmap3_Ma_ORF 529 ATCATGGCCAACAGTAATCATCGTTTTAATCTGGCCCCCTATTTTTGGTTCAATCAGTGCTTATTA 594

Pjmap3_Ma_gen 529 ATCATGGCCAACAGTAATCATCGTTTTAATCTGGCCCCCTATTTTTGGTTCAATCAGTGCTTATTA 594

529 ****************************************************************** 594

_____________________intron 1 __________________

Pjmap3_Cissé_ORF 525 TTCAG------------------------------------------------CTAAAGTAATATA 542

Pjmap3_Cissé_gen 525 TTCAG**GT**ACACCATTTGCACGGTTTATAATAAATGTAT**T**T**A**TCATTTTATT**AG**CTAAAGTAATATA 590

Pjmap3_Ma_ORF 595 TTCAG**GT**ACACCATTTGCACAGTTTATAATAAATGTAT**T**T**A**TCATTTTATT**AG**CTAAAGTAATATA 660

Pjmap3_Ma_gen 595 TTCAG**GT**ACACCATTTGCACAGTTTATAATAAATGTAT**T**T**A**TCATTTTATT**AG**CTAAAGTAATATA 660

595 ***** ************* 660

Pjmap3_Cissé_ORF 543 CCTATATTTCAAAAAACAAAAAGAATTTCAAAATGTTTTAAGAGATTCTAAAACATCTATGACACT 608

Pjmap3_Cissé_gen 591 CCTATATTTCAAAAAACAAAAAGAATTTCAAAATGTTTTAAGAGATTCTAAAACATCTATGACACT 656

Pjmap3_Ma_ORF 661 CCTATATTTCAAAAAACAAAAAGAATTTCAAAATGTTTTAAGAGATTCTAAAACATCTATGACACT 726

Pjmap3_Ma_gen 661 CCTATATTTCAAAAAACAAAAAGAATTTCAAAATGTTTTAAGAGATTCTAAAACATCTATGACACT 726

661 ****************************************************************** 726

Pjmap3_Cissé_ORF 609 TTCTAGATTTGTACGTCTTATAGGCATATGCTCCCTTTTAGTCACTGTTTATTTACCATTAAATAT 674

Pjmap3_Cissé_gen 657 TTCTAGATTTGTACGTCTTATAGGCATATGCTCCCTTTTAGTCACTGTTTATTTACCATTAAATAT 722

Pjmap3_Ma_ORF 727 TTCTAGATTTGTACGTCTTATAGGCATATGCTCCCTTTTAGTCACTGTTTATTTACCATTAAATAT 792

Pjmap3_Ma_gen 727 TTCTAGATTTGTACGTCTTATAGGCATATGCTCCCTTTTAGTCACTGTTTATTTACCATTAAATAT 792

727 ****************************************************************** 792

Pjmap3_Cissé_ORF 675 TTACATGTTATATACGAATATATTTCTAATTATTCAAAGCAAAATAAACTATTCATGGGACCATGT 740

Pjmap3_Cissé_gen 723 TTACATGTTATATACGAATATATTTCTAATTATTCAAAGCAAAATAAACTATTCATGGGACCATGT 788

Pjmap3_Ma_ORF 793 TTACATGTTATATACGAATATATTTCTAATTATTCAAAGCAAAATAAACTATTCATGGGACCATGT 858

Pjmap3_Ma_gen 793 TTACATGTTATATACGAATATATTTCTAATTATTCAAAGCAAAATAAACTATTCATGGGACCATGT 858

793 ****************************************************************** 858

Pjmap3_Cissé_ORF 741 TCATCAATGGGGTCATGGTATTGCTTATTTGAAAAATGATAAAATATCTTTTAATCTATGGCTTAT 806

Pjmap3_Cissé_gen 789 TCATCAATGGGGTCATGGTATTGCTTATTTGAAAAATGATAAAATATCTTTTAATCTATGGCTTAT 854

Pjmap3_Ma_ORF 859 TCATCAATGGGGTCATGGTATTGCTTATTTGAAAAATGATAAAATATCTTTTAATCTATGGCTTAT 924

Pjmap3_Ma_gen 859 TCATCAATGGGGTCATGGTATTGCTTATTTGAAAAATGATAAAATATCTTTTAATCTATGGCTTAT 924

859 ****************************************************************** 924

Pjmap3_Cissé_ORF 807 ACCGTCAAATAGTATTGTTGTTTTCATTTTTTTTGGTATGGGAAGTGATGCCATTGTCATGTATAA 872

Pjmap3_Cissé_gen 855 ACCGTCAAATAGTATTGTTGTTTTCATTTTTTTTGGTATGGGAAGTGATGCCATTGTCATGTATAA 920

Pjmap3_Ma_ORF 925 ACCGTCAAATAGTATTGTTGTTTTCATTTTTTTTGGTATGGGAAGTGATGCCATTGTCATGTATAA 990

Pjmap3_Ma_gen 925 ACCGTCAAATAGTATTGTTGTTTTCATTTTTTTTGGTATGGGAAGTGATGCCATTGTCATGTATAA 990

925 ****************************************************************** 990

Pjmap3_Cissé_ORF 873 AGAAGTGGCAAGAAAACTGTATATAATTAAATTTTTTGATTTCTTTAAAAGAATGTTCAAAAGAAA 938

Pjmap3_Cissé_gen 921 AGAAGTGGCAAGAAAACTGTATATAATTAAATTTTTTGATTTCTTTAAAAGAATGTTCAAAAGAAA 986

Pjmap3_Ma_ORF 991 AGAAGTGGCAAGAAAACTGTATATAATTAAATTTTTTGATTTCTTTAAAAGAATGTTCAAAAGAAA 1056

Pjmap3_Ma_gen 991 AGAAGTGGCAAGAAAACTGTATATAATTAAATTTTTTGATTTCTTTAAAAGAATGTTCAAAAGAAA 1056

991 ****************************************************************** 1056

___

Pjmap3_Cissé_ORF 939 GACTCAGGATGTCAGTAACAAAGACTATTATAACAGCTATAATTTCGAAAAATCATTAGACAG--- 999

Pjmap3_Cissé_gen 987 GACTCAGGATGTCAGTAACAAAGACTATTATAACAGCTATAATTTCGAAAAATCATTAGACAG**GT**A 1052

Pjmap3_Ma_ORF 1057 GACTCAGGATGTCAGTAACAAAGACTATTATAACAGCTATAATTTCGAAAAATCATTAGACAG--- 1117

Pjmap3_Ma_gen 1057 GACTCAGGATGTCAGTAACAAAGACTATTATAACAGCTATAATTTCGAAAAATCATTAGACAG**GT**A 1122

1057 *************************************************************** 1122

____________intron 2____________________

Pjmap3_Cissé_ORF 1000 ----------------------------------------ATGTCCACCATTGTTTTATAACCAAG 1027

Pjmap3_Cissé_gen 1053 AGACACATCAGTTCTAAGGTATCTTT**T**T**A**CAGGCTTCC**AG**ATGTCCACCATTGTTTTATAACCAAG 1118

Pjmap3_Ma_ORF 1118 ----------------------------------------ATGTCCACCATTGTTTTATAACCAAG 1145

Pjmap3_Ma_gen 1123 AGACACATCAGTTCTAAGGTATCTTT**T**T**A**CAGGCTTCC**AG**ATGTCCACCATTGTTTTATAACCAAG 1188

1123 ************************** 1188

Pjmap3_Cissé_ORF 1028 TACGCGATGCACAGATTATAGAAAACAACTCTTTTAGCGACCATCCTGCAATCCCTCCAATATACA 1093

Pjmap3_Cissé_gen 1119 TACGCGATGCACAGATTATAGAAAACAACTCTTTTAGCGACCATCCTGCAATCCCTCCAATATACA 1184

Pjmap3_Ma_ORF 1146 TACGCGATGCACAGATTATAGAAAACAACTCTTTTAGCGACCATCCTGCAATCCCTCCAATATACA 1211

Pjmap3_Ma_gen 1189 TACGCGATGCACAGATTATAGAAAACAACTCTTTTAGCGACCATCCTGCAATCCCTCCAATATACA 1254

1189 ****************************************************************** 1254

Pjmap3_Cissé_ORF 1094 TGGAACACAGCAAACCATACACCTTTACAGAT---------------------------------- 1122

Pjmap3_Cissé_gen 1185 TGGAACACAGCAAACCATACACCTTTACAGAT---------------------------------- 1213

Pjmap3_Ma_ORF 1212 TGGAACACAGCAAACCATACACCTTTACAGATGTACCTATTTATTCCCATAGCAAAAACACCTATT 1277

Pjmap3_Ma_gen 1255 TGGAACACAGCAAACCATACACCTTTACAGATGTACCTATTTATTCCCATAGCAAAAACACCTATT 1320

1255 ******************************** 1320

Pjmap3_Cissé_ORF 1123 -------------------------------------------------- 1125

Pjmap3_Cissé_gen 1214 -------------------------------------------------- 1216

Pjmap3_Ma_ORF 1278 CCATGCCCTTTGAAAAATACCAATATGAATTTAGAAATGATAAAATATAA 1327

Pjmap3_Ma_gen 1321 CCATGCCCTTTGAAAAATACCAATATGAATTTAGAAATGATAAAATATAA 1370

1321 1370
