# Supplementary material for: Expression and Immunostaining Analyses Suggest that Pneumocystis Primary Homothallism Involves Trophic Cells Displaying Both Plus and Minus Pheromone Receptors
Source: mBio. 2019 Jul 9;10(4):e01145-19. doi: 10.1128/mBio.01145-19 (PMC6747714; doi:10.1128/mBio.01145-19)
Supplement: FIG S2 [file mBio.01145-19-sf002.docx]

**Fig. S2**

**A**

Pmmam2_ORF 1 ATGGCATTTTCTCCAACAAACCAGACAATATTTCTTAAGAACTCTAAAGGAGAAACAATTCCATTTTTATTATCGGAT 78

Pmmam2_gen 1 ATGGCATTTTCTCCAACAAACCAGACAATATTTCTTAAGAACTCTAAAGGAGAAACAATTCCATTTTTATTATCGGAT 78

1 ****************************************************************************** 78

Pmmam2_ORF 79 TTCGATGAATTTTCACTCTCTAGAGCTCAAACGTCTATGATATTTTCAGCACAATGTGCAATGAGCTTATTATTGGCA 156

Pmmam2_gen 79 TTCGATGAATTTTCACTCTCTAGAGCTCAAACGTCTATGATATTTTCAGCACAATGTGCAATGAGCTTATTATTGGCA 156

79 ****************************************************************************** 156

Pmmam2_ORF 157 CTTGTCCTTATGTTGACATCAAAAAGAGAAAAAAGAAAAACATTGCTTTTCTTTTTAAACATAGGTGGATTAGTAACA 234

Pmmam2_gen 157 CTTGTCCTTATGTTGACATCAAAAAGAGAAAAAAGAAAAACATTGCTTTTCTTTTTAAACATAGGTGGATTAGTAACA 234

157 ****************************************************************************** 234

Pmmam2_ORF 235 GTATTTATAAGAGCATGTCTTCAATGTGCTTATTTGTCAGGCACCTGGGTAAGCTATAGTGTCCAATTTCTTGGAGAA 312

Pmmam2_gen 235 GTATTTATAAGAGCATGTCTTCAATGTGCTTATTTGTCAGGCACCTGGGTAAGCTATAGTGTCCAATTTCTTGGAGAA 312

235 ****************************************************************************** 312

Pmmam2_ORF 313 TTTGAATTATTGTCGCAAAAAGACTTTTATATATCAATTATCGCATCATGTATTCCAATTTTTATTATTTTGTTCATC 390

Pmmam2_gen 313 TTTGAATTATTGTCGCAAAAAGACTTTTATATATCAATTATCGCATCATGTATTCCAATTTTTATTATTTTGTTCATC 390

313 ****************************************************************************** 390

Pmmam2_ORF 391 GAACTTTCTCTTCTCATTCAAATTAGAGTAGTATATGCAACTGATAAAAGATTACAGATACCATTGACAATATTTTTT 468

Pmmam2_gen 391 GAACTTTCTCTTCTCATTCAAATTAGAGTAGTATATGCAACTGATAAAAGATTACAGATACCATTGACAATATTTTTT 468

391 ****************************************************************************** 468

Pmmam2_ORF 469 TCTATAATAATAATTATCGTTATAACCTTTTGGATTTTAGCTGCTGTTCAAAACTCAATGGCAGTTTTATCTCAAACA 546

Pmmam2_gen 469 TCTATAATAATAATTATCGTTATAACCTTTTGGATTTTAGCTGCTGTTCAAAACTCAATGGCAGTTTTATCTCAAACA 546

469 ****************************************************************************** 546

Pmmam2_ORF 547 CATTTTGGACATAGCGGTGTATGGGGCGCACCCTGGCCTTATACAGTAGCACGCATATCTTTTGCTTTTAGTATATTT 624

Pmmam2_gen 547 CATTTTGGACATAGCGGTGTATGGGGCGCACCCTGGCCTTATACAGTAGCACGCATATCTTTTGCTTTTAGTATATTT 624

547 ****************************************************************************** 624

Pmmam2_ORF 625 ATAGGATGTATTGTTTTTATTTATAAATTGCTTATCACCATTTATCGAAGACATAAAATGGGAGTCAAAGAATTTGGA 702

Pmmam2_gen 625 ATAGGATGTATTGTTTTTATTTATAAATTGCTTATCACCATTTATCGAAGACATAAAATGGGAGTCAAAGAATTTGGA 702

625 ****************************************************************************** 702

________________intron 1_____

Pmmam2_ORF 703 CCAATACAAATTATATTTATTATGAGCTGTCAAACATTGATCATCCCTG----------------------------- 750

Pmmam2_gen 703 CCAATACAAATTATATTTATTATGAGCTGTCAAACATTGATCATCCCTG**GT**ATTTTTTGTCACATTCTGTTTTATTTC 780

703 ************************************************* 780

________________

Pmmam2_ORF 751 ----------------CCATTCTCATTCTTATCGATTTTGGAGTAAAAATAACAGGTTTTAGCTCATTAACTCAAGCG 813

Pmmam2_gen 781 **TAA**TA**T**T**A**TTTTAT**AG**CCATTCTCATTCTTATCGATTTTGGAGTAAAAATAACAGGTTTTAGCTCATTAACTCAAGCG 858

781 *************************************************************** 858

Pmmam2_ORF 814 CTCGTCGTAATGTCTTTACCTTTATCTTCTCTTTGGGCATCATCTAAAGTCGAAAAAAATAAAAACAACGTAACACCA 891

Pmmam2_gen 859 CTCGTCGTAATGTCTTTACCTTTATCTTCTCTTTGGGCATCATCTAAAGTCGAAAAAAATAAAAACAACGTAACACCA 936

859 ****************************************************************************** 936

Pmmam2_ORF 892 ACATATTATAAAGATATGAAGAGCATTGGAGATTATAGTATTGAAAGCACACCGAGTTCATTCACTAAACCATCTTAT 969

Pmmam2_gen 937 ACATATTATAAAGATATGAAGAGCATTGGAGATTATAGTATTGAAAGCACACCGAGTTCATTCACTAAACCATCTTAT 1014

937 ****************************************************************************** 1014

Pmmam2_ORF 970 ATAGGGTTTAGAAAACCATCGTATTTCTCGGAATATTCTAAAAGTCCTTTCTATGATGAGTATTTTGATGATAATGGG 1047

Pmmam2_gen 1015 ATAGGGTTTAGAAAACCATCGTATTTCTCGGAATATTCTAAAAGTCCTTTCTATGATGAGTATTTTGATGATAATGGG 1092

1015 ****************************************************************************** 1092

Pmmam2_ORF 1048 TCTAAACTTGATATACTAGTGGAAAAATCTCTAAATGTGTTTCCAGAAAAGAAATAG 1105

Pmmam2_gen 1093 TCTAAACTTGATATACTAGTGGAAAAATCTCTAAATGTGTTTCCAGAAAAGAAATAG 1150

1093 ********************************************************* 1150

**B**

Pmmap3_ORF 1 ATGGGAGAAGTGTTTTATATCTTTTTTTGCTTGATTGGATTTTTATGTTCAATTATACCTTCTATCTGGCACTGGAAA 78

Pmmap3_gen 1 ATGGGAGAAGTGTTTTATATCTTTTTTTGCTTGATTGGATTTTTATGTTCAATTATACCTTCTATCTGGCACTGGAAA 78

1 ****************************************************************************** 78

Pmmap3_ORF 79 TATCGAAATGTCGCGCCTTTATGTCTTATTTTTTGGATATCTTCAACTAATTTAATATATTTCATTAATTCTATCATC 156

Pmmap3_gen 79 TATCGAAATGTCGCGCCTTTATGTCTTATTTTTTGGATATCTTCAACTAATTTAATATATTTCATTAATTCTATCATC 156

79 ****************************************************************************** 156

Pmmap3_ORF 157 TGGTTTAACGGATCTAAATCAACATATCGTGGTGATCTATATTGTGACATTGTGACTAAGCTTATACTCGGATCGGTT 234

Pmmap3_gen 157 TGGTTTAACGGATCTAAATCAACATATCGTGGTGATCTATATTGTGACATTGTGACTAAGCTTATACTCGGATCGGTT 234

157 ****************************************************************************** 234

Pmmap3_ORF 235 ACTGGAGAATTAGGTGCTACTGTTGCTATTACACATTACCTTTCGAAAATTATGAAGTCTTCATATTCATCTATCCAG 312

Pmmap3_gen 235 ACTGGAGAATTAGGTGCTACTGTTGCTATTACACATTACCTTTCGAAAATTATGAAGTCTTCATATTCATCTATCCAG 312

235 ****************************************************************************** 312

Pmmap3_ORF 313 TCTAAAATAACTCGTAGAAATCAAGCAATAGAAGATATTCTTTTTAGTTTTACATGTCCTATTATAATAATGTCTTTA 390

Pmmap3_gen 313 TCTAAAATAACTCGTAGAAATCAAGCAATAGAAGATATTCTTTTTAGTTTTACATGTCCTATTATAATAATGTCTTTA 390

313 ****************************************************************************** 390

Pmmap3_ORF 391 CATTATATCGTTCAGCCAGCAAGGTATGTAATCGATGGAATTAGTGGATGCATGCCATGGACGGATCGATCATGGCTA 468

Pmmap3_gen 391 CATTATATCGTTCAGCCAGCAAGGTATGTAATCGATGGAATTAGTGGATGCATGCCATGGACGGATCGATCATGGCTA 468

391 ****************************************************************************** 468

_________________

Pmmap3_ORF 469 GCTGTGATTATTGTTTTATTATGGCCTCCTGTATTTGGTAGTATCAGTGCTTACTATTCAG----------------- 528

Pmmap3_gen 469 GCTGTGATTATTGTTTTATTATGGCCTCCTGTATTTGGTAGTATCAGTGCTTACTATTCAG**GT**ATATTCTTCGTA**TGA** 546

469 ************************************************************* 546

__intron 1 _________________

Pmmap3_ORF 529 ----------------------------TTAAAGTAATTATTTCATACTTCAAAAAGCGAAATGAATTTCAAACTATT 579

Pmmap3_gen 547 TTTATAA**T**A**A**ATGTATTCATTTTATT**AG**TTAAAGTAATTATTTCATACTTCAAAAAGCGAAATGAATTTCAAACTATT 624

547 ************************************************** 624

Pmmap3_ORF 580 TTAAAAGATTCAAAATCGTCTATGACTTTATCAAGATTCATACGTCTTATAGGCTTATCATCTCTAATAATAGCCATT 657

Pmmap3_gen 625 TTAAAAGATTCAAAATCGTCTATGACTTTATCAAGATTCATACGTCTTATAGGCTTATCATCTCTAATAATAGCCATT 702

625 ****************************************************************************** 702

Pmmap3_ORF 658 TACTTGCCACTTAATATTTACTTGTTAGCTATAAACATAGCCCAGATTATTAAAAGCAACATCAAATATTCATGGTCA 735

Pmmap3_gen 703 TACTTGCCACTTAATATTTACTTGTTAGCTATAAACATAGCCCAGATTATTAAAAGCAACATCAAATATTCATGGTCA 780

703 ****************************************************************************** 780

Pmmap3_ORF 736 GATGTTCATAATTGGAATTCTAGTATTTTTTATCTTCCAAAAAGCAACATGCCTTTCAATCGCTGGCTTTCGCCATCT 813

Pmmap3_gen 781 GATGTTCATAATTGGAATTCTAGTATTTTTTATCTTCCAAAAAGCAACATGCCTTTCAATCGCTGGCTTTCGCCATCT 858

781 ****************************************************************************** 858

Pmmap3_ORF 814 AATGGCATTATTGTTTTTATCTTTTTTGGTATGGGTAATGATGCAATACTCATGTATAAAGAAATAGCAAGAAAATTG 891

Pmmap3_gen 859 AATGGCATTATTGTTTTTATCTTTTTTGGTATGGGTAATGATGCAATACTCATGTATAAAGAAATAGCAAGAAAATTG 936

859 ****************************************************************************** 936

Pmmap3_ORF 892 TACATTACTCAATTTTTCCATTTTGTCCAAAAGAAAATTTTCAAAAAAAAAACAGAAGATAACAAAAATTCACAGGAT 969

Pmmap3_gen 937 TACATTACTCAATTTTTCCATTTTGTCCAAAAGAAAATTTTCAAAAAAAAAACAGAAGATAACAAAAATTCACAGGAT 1014

937 ****************************************************************************** 1014

___________________intron 2_____________

Pmmap3_ORF 970 TATTATAATAGCTATAGTTTTGAAAAATCATTAAACAG---------------------------------------- 1007

Pmmap3_gen 1015 TATTATAATAGCTATAGTTTTGAAAAATCATTAAACAG**GT**AAGATAAATCAATCTTTTAATATCCTTTTCC**T**T**A**CAAA 1092

1015 ************************************** 1092

_________

Pmmap3_ORF 1008 ---------TTGTCCTCCATTATTTTATAATCAAACACGAGACGTAAGAATTCTTGAAAATGGTTCTTTAAATGATTA 1076

Pmmap3_gen 1093 TATATTT**AG**TTGTCCTCCATTATTTTATAATCAAACACGAGACGTAAGAATTCTTGAAAATGGTTCTTTAAATGATTA 1170

1093 ********************************************************************* 1170

Pmmap3_ORF 1077 TTCTTCTCCTCCTATTTATACAGATCATGACAAGTACAACTTAGATTTATCAATCTATAATCAGTATTACAGAGACAA 1154

Pmmap3_gen 1171 TTCTTCTCCTCCTATTTATACAGATCATGACAAGTACAACTTAGATTTATCAATCTATAATCAGTATTACAGAGACAA 1248

1171 ****************************************************************************** 1248

Pmmap3_ORF 1155 TTCTAATATAAATAATAAATACGGGCCTCGCAAATAA 1191

Pmmap3_gen 1249 TTCTAATATAAATAATAAATACGGGCCTCGCAAATAA 1285

1249 ************************************* 1285
