# Supplementary figures and images for: Expression and Immunostaining Analyses Suggest that Pneumocystis Primary Homothallism Involves Trophic Cells Displaying Both Plus and Minus Pheromone Receptors
Source: mBio. 2019 Jul 9;10(4):e01145-19. doi: 10.1128/mBio.01145-19 (PMC6747714; doi:10.1128/mBio.01145-19)

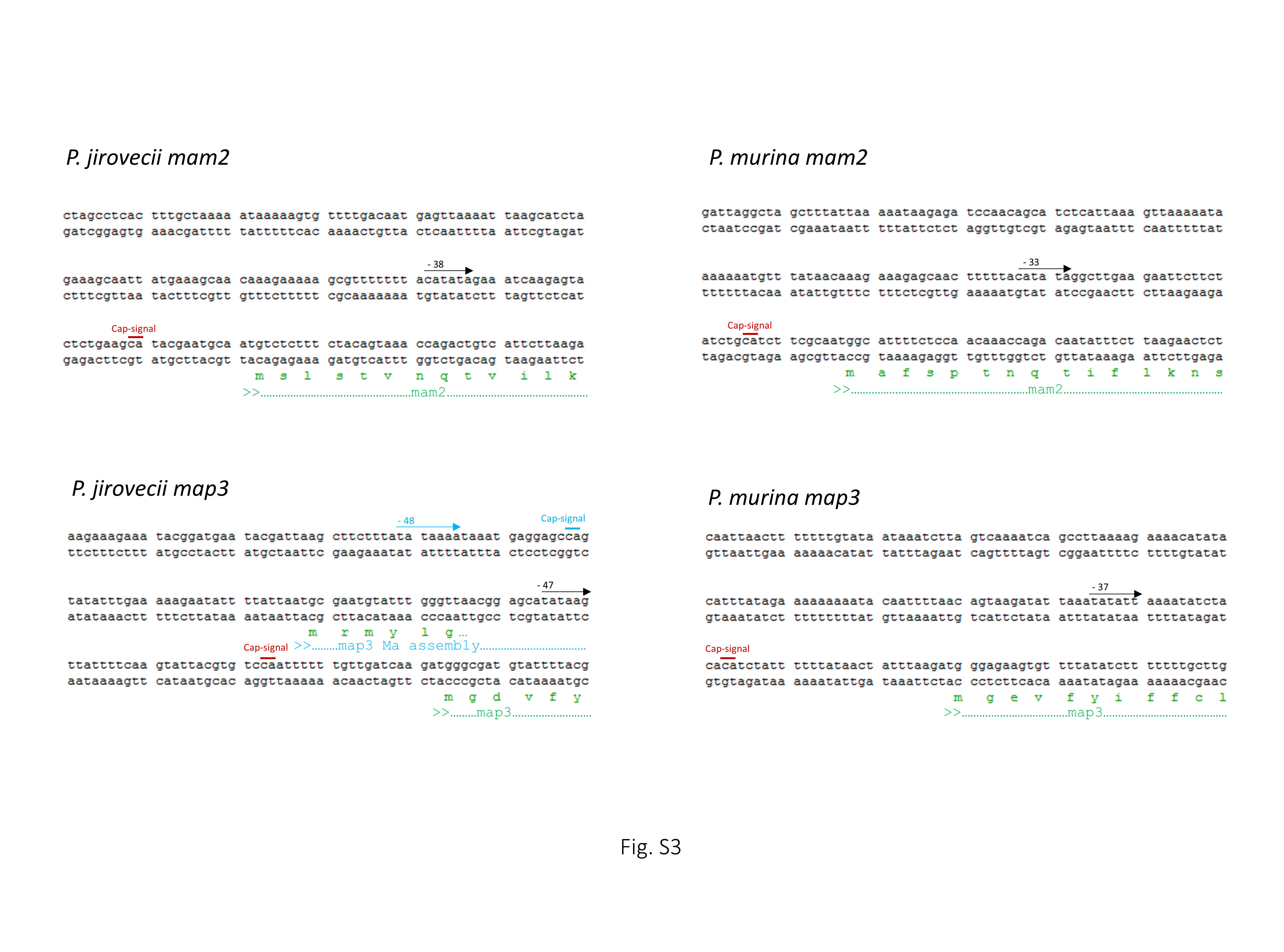

Supplement: FIG S3 [file mBio.01145-19-sf003.tif]
