# Supplementary material for: Expression and Immunostaining Analyses Suggest that Pneumocystis Primary Homothallism Involves Trophic Cells Displaying Both Plus and Minus Pheromone Receptors
Source: mBio. 2019 Jul 9;10(4):e01145-19. doi: 10.1128/mBio.01145-19 (PMC6747714; doi:10.1128/mBio.01145-19)
Supplement: FIG S4 [file mBio.01145-19-sf004.pdf]

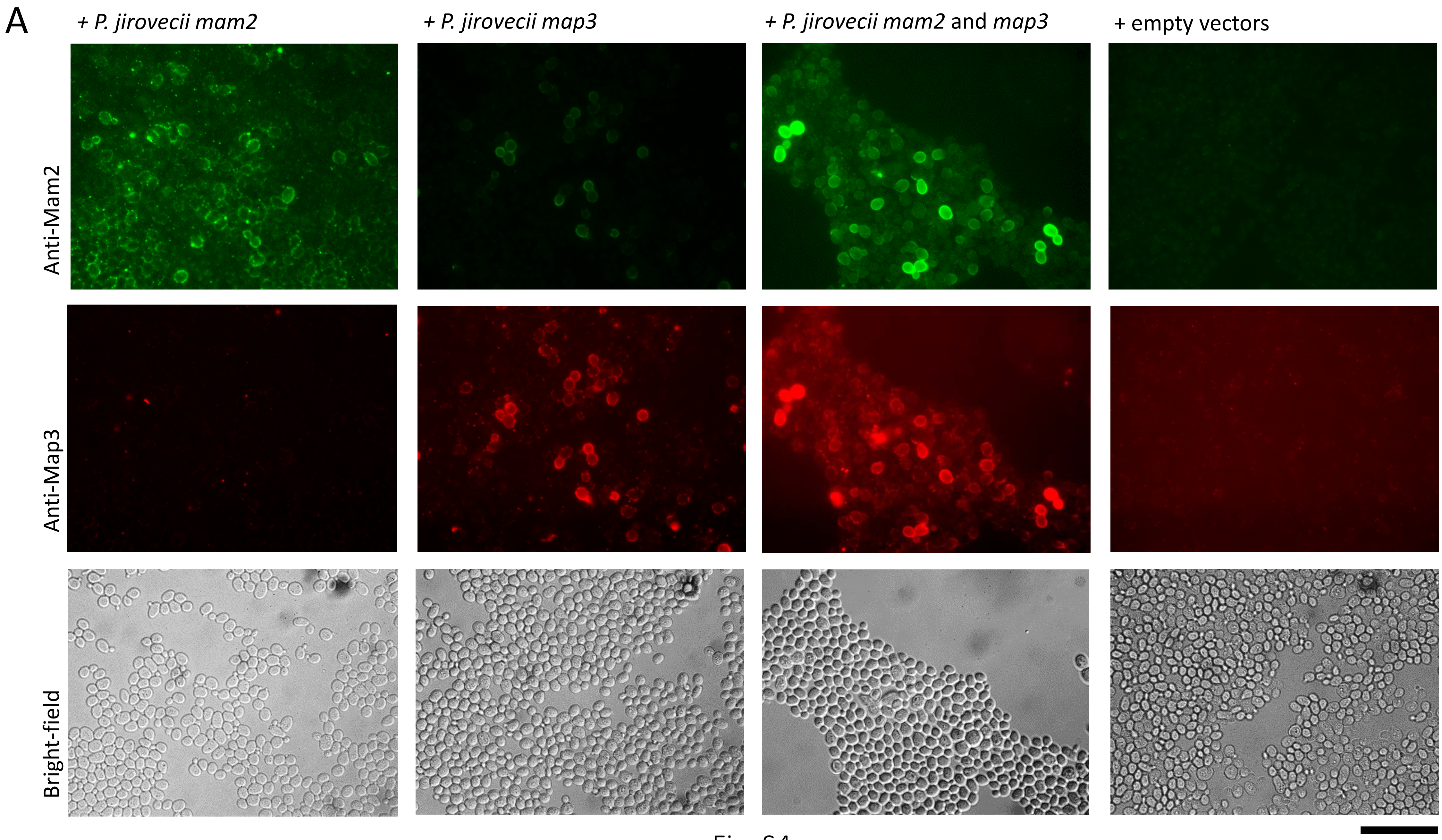
A
+ P. jirovecii mam2
+ P. jirovecii map3
+ P. jirovecii mam2 and map3
+ empty vectors
Anti-Mam2
Anti-Map3
Bright-field

Fig. S4

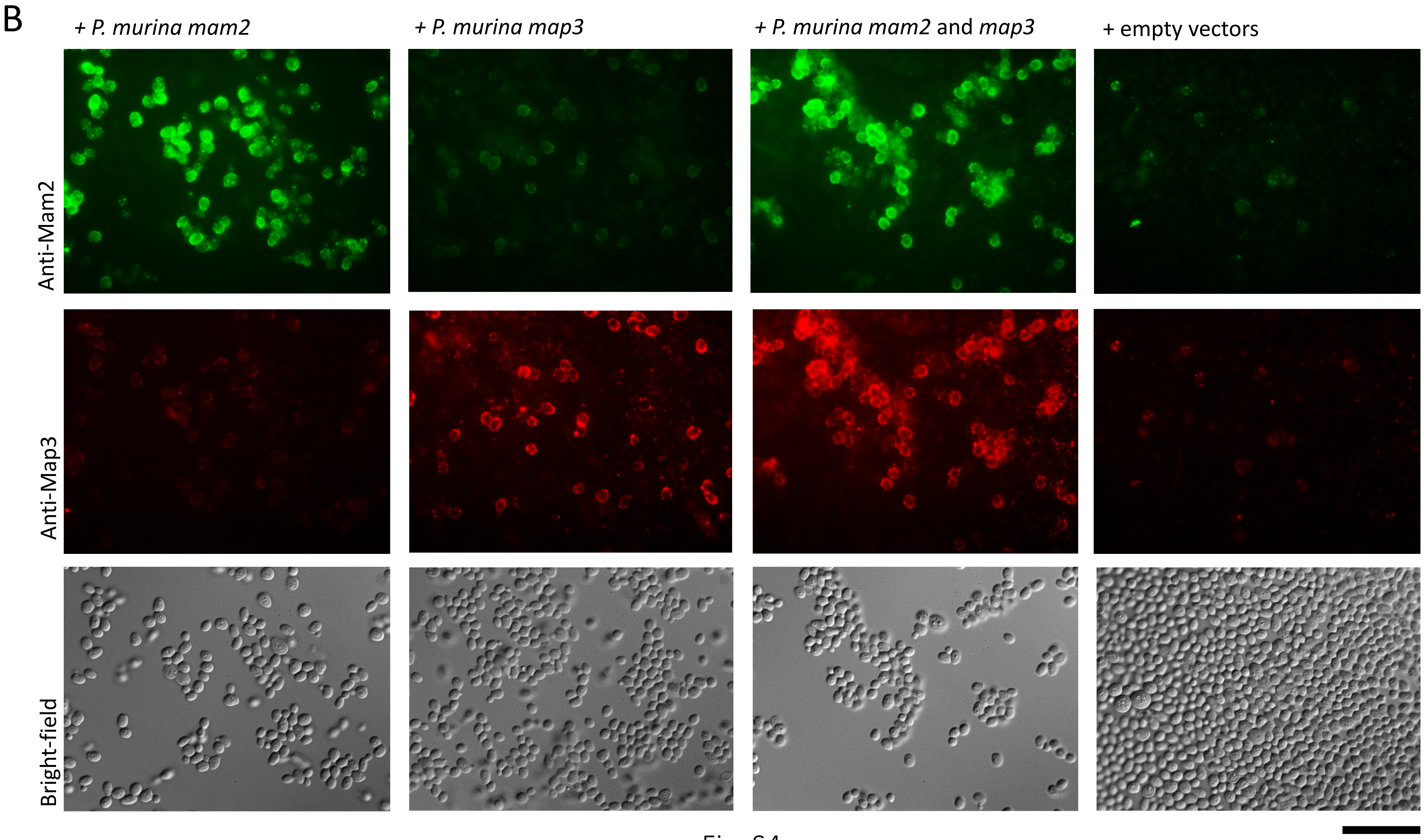
B
+ P. murina mam2
+ P. murina map3
+ P. murina mam2 and map3
+ empty vectors
Anti-Mam2
Anti-Map3
Bright-field

Fig. S4
