# Supplementary material for: Expression and Immunostaining Analyses Suggest that Pneumocystis Primary Homothallism Involves Trophic Cells Displaying Both Plus and Minus Pheromone Receptors
Source: mBio. 2019 Jul 9;10(4):e01145-19. doi: 10.1128/mBio.01145-19 (PMC6747714; doi:10.1128/mBio.01145-19)
Supplement: TABLE S1 [file mBio.01145-19-st001.docx]

| Target^a^ | Primer name | Primer 5'-3' | PCR product size (bp) with/without intron | Description | Annealing temperature (°C) |
| --- | --- | --- | --- | --- | --- |
| *Pj β-tub* | Pj-Btub-for | TTTTCAGTGGTTCCCTCACC | 150/103 | Amplification of internal fragment from position 677 to 827 of genomic DNA, this region encompasses the intron no. 6 of 47 bps | 58 |
|  | Pj-Btub-rev | AGAATGTTTCATCGGAATTTTCA |  |  |  |
| *Pj mam2* | Pj-mam2-for | GCTGCTATTCAAAATTCAATGG | 169/140 | Amplification of internal fragment from position 508 to 677 of genomic DNA, this region encompasses the intron no. 1 of 30 bps | 58 |
|  | Pj-mam2-rev | TGTCTACGGTAAATTGCG |  |  |  |
| *Pj map3* | Pj-map3-for | TCTGGCCCCCTATTTTTGG | 186/139 | Amplification of internal fragment from position 488 to 674 of genomic DNA, this region encompasses the intron no. 1 of 48 bps | 58 |
|  | Pj-map3-rev | AGACGTACAAATCTAGAAAGTGTC |  |  |  |
| *Pm β-tub* | Pm-Btub-for | CATACAATGCGACGCTTTCT | 156/109 | Amplification of internal fragment from position 878 to 1034 of genomic DNA, this region encompasses the intron no. 6 of 47 bps | 60 |
|  | Pm-Btub-rev | GGAAGCTTTAATGTTCGCATAC |  |  |  |
| *Pm mam2* | Pm-mam2-for | ATTATGAGCTGTCAAACATTG | 148/104 | Amplification of internal fragment from position 721 to 869 of genomic DNA, this region encompasses the unique intron of 45 bps | 56 |
|  | Pm-mam2-rev | ATTACGACGAGCGCTTGAG |  |  |  |
| *Pm map3* | Pm-map3-for | CGATCATGGCTAGCTGTG | 153/109 | Amplification of internal fragment from position 457 to 610 of genomic DNA, this region encompasses the intron no. 1 of 45 bps | 56 |
|  | Pm-map3-rev | CATTTCGCTTTTTGAAGTATG |  |  |  |
| *Pm matMc* | Pm-matMc-for | GAATCCTCCACGACCACCTA | 151 (no intron) | Amplification of internal fragment from position 234 to 384 | 60 |
|  | Pm-matMc-rev | TCGCTGTTTTACAGCTGGTG |  |  |  |
| *Pm matMi* | Pm-matMi-for | TGTTTACCATTTCACCTTCACC | 100 (no intron) | Amplification of internal fragment from position 2 to 101 | 60 |
|  | Pm-matMi-rev | TTTTCACTGGCTAATGCATGG |  |  |  |
| *Pm matPi* | Pm-matPi-for | CAACAAGGAATTGTCGGAGAC | 164 (no intron) | Amplification of internal fragment from position 171 to 334 | 60 |
|  | Pm-matPi-rev | TCCGACATAAATCCGACAGA |  |  |  |

**Table S1**. PCR primers and conditions.

^a^ *Pj, P. jirovecii; Pm, P. murina.*
